# The Immunomodulatory Effects of Porcupine Bezoar on Cyclophosphamide-Induced Immunosuppression in Rats

**DOI:** 10.3390/ph19040563

**Published:** 2026-04-01

**Authors:** Ji Li, Wenbo Gao, Kien-Seng Lim, Song Lei, Zhipeng Chen, Xiao-Qing Sim, Qinqiang Long, Xue Xiao

**Affiliations:** 1 Guangdong Metabolic Diseases Research Center of Integrated Chinese and Western Medicine, Guangdong Pharmaceutical University, Guangzhou 510006, China; 2 Miracle Medicine Sdn Bhd, F-01-10, Level 1, Block F, Sunway Geo Avenue, Jalan Lagoon Selatan, Sunway South Quay, Bandar Sunway, Subang Jaya 47500, Malaysia; 3 Jiyuan Neurohealth Industry Research Institute of Guangdong Pharmaceutical University, Guangzhou 510006, China

**Keywords:** porcupine bezoar, immunosuppression, cyclophosphamide, immunomodulation, gut microbiota, metabolomics

## Abstract

**Background/Objectives**: Immunosuppression is a serious side effect of chemotherapeutic agents such as cyclophosphamide (CTX) and significantly increases the risk of infection in patients. Porcupine (*Hystrix brachyura*) bezoar (PB), a traditional medicine derived from the Hystrix brachyura species of porcupine, is renowned for its antioxidant and anti-inflammatory properties. However, its immunomodulatory potential has not been adequately investigated. This study aimed to systematically evaluate the protective effects of PB against CTX-induced immunosuppression and the underlying mechanisms in a rat model. **Methods**: An immunosuppression model was established in rats through the injection of CTX. The effects of PB on immune function were evaluated through the measurement of serum immunoglobulin (IgA and IgG) and pro-inflammatory cytokine (IL-6 and TNF-α) levels, as well as through a histopathological examination of immune organs. The mechanisms were further elucidated by analysing changes in serum metabolites and gut microbiota composition using integrated metabolomics and 16S rRNA sequencing. **Results**: Treatment with PB significantly alleviated CTX-induced immunosuppression, as demonstrated by elevated serum levels of IgA and IgG and reduced concentrations of IL-6 and TNF-α. PB also improved the architecture of spleen and thymus tissues. Metabolomic analysis revealed that PB regulated glycerophospholipid metabolism and steroid hormone biosynthesis, thereby reducing pro-inflammatory metabolites such as prostaglandin F2α. Furthermore, PB modulated the gut microbiota, increasing the abundance of beneficial bacteria (e.g., *Bacteroidota* and *Lachnospiraceae*) and decreasing that of harmful bacteria (e.g., *Romboutsia* and *Clostridium sensu stricto*). **Conclusions**: This study demonstrates that PB can effectively counteract CTX-induced immunosuppression in rats. This immunomodulatory effect is linked to changes in the gut microbiota and the regulation of specific metabolic pathways. These findings provide a scientific basis for the potential use of PB as an immunoadjuvant therapy, offering new insights into the mechanisms of traditional medicines.

## 1. Introduction

The immune system, composed of immune organs, cells, and immune-active molecules, plays essential roles in immune surveillance, defense, and homeostasis [1]. It is crucial for maintaining health, orchestrating immune responses, and protecting the body against external pathogens [2,3]. Cancer, which arises from the malignant transformation and uncontrolled proliferation of cells, presents significant challenges to the immune system [4]. Tumors can directly suppress immune responses via immune evasion mechanisms and by remodeling the tumor microenvironment, leading to immune dysfunction and promoting tumor progression and metastasis [5].

Chemotherapy is a widely used clinical treatment for cancer that works by administering chemical agents to inhibit tumour cell proliferation. However, these drugs often lack specificity for tumour cells, resulting in widespread damage to normal tissues and cells. While this non-selective action is effective in reducing tumour growth, it can also impair the immune system through mechanisms such as bone marrow suppression and gut microbiota dysbiosis, leading to severe toxic side effects [6,7,8]. Consequently, mitigating chemotherapy-induced immunosuppression and enhancing immune function have become critical areas of research in cancer therapy. According to Global Cancer Statistics 2022: GLOBOCAN estimates of incidence and mortality worldwide for 36 cancer types in 185 countries, approximately 20 million new cancer cases were diagnosed globally in 2022, resulting in 9.6 million deaths [9]. The report predicts that cancer incidence and mortality rates will continue to rise over the next two decades due to population growth and ageing. Of particular concern is the growing cancer burden in China, where the ageing population could cause cancer mortality rates to exceed the global average [10]. Despite the widespread use of various anticancer drugs, their immunosuppressive effects continue to severely impact patient outcomes and quality of life [11]. Immunosuppression is widely recognised as a key factor in the onset, progression and metastasis of tumours, and further complicate cancer treatment [12,13].

Cyclophosphamide (CTX) is a clinically established alkylating chemotherapeutic agent that has been widely used not only in cancer treatment but also as a classical inducer of experimental immunosuppression [14,15,16]. Its usefulness in this context is mainly attributable to its well-characterized myelosuppressive and immunosuppressive effects, including inhibition of DNA replication and depletion of rapidly proliferating immune cells such as lymphocytes. Consequently, the CTX-induced immunosuppression model reflects key features of chemotherapy-related immune dysfunction and provides a practical platform for evaluating candidate immunoprotective interventions. The chemical structure of CTX is shown in Supplementary File Figure S1, and its molecular formula is C_7_H_15_Cl_2_N_2_O_2_P [17,18,19].

The porcupine bezoar (PB), a traditional medicinal plant, has recently attracted attention in Chinese medicinal research. Derived from the porcupine, a wild herbivorous animal native to Southeast Asia, it is known for its unique immune-boosting properties. Traditional medicine holds that it can clear heat, detoxify the body, reduce inflammation, and boost immune function [20]. Historical texts such as the ‘*Compendium of Materia Medica*’ and ‘*Luchuan Materia Medica*’ describe the medicinal value of various porcupine parts, including the belly, meat, and quills. These parts have been used to treat conditions such as oedema, jaundice, and beriberi [21,22,23]. Although modern pharmacological research on porcupine bezoar remains limited, preliminary studies suggest that it is rich in amino acids, natural antioxidants (e.g., polyphenolic compounds) and immune-modulating factors (e.g., polysaccharides and saponins). These bioactive compounds may enhance antioxidant capacity by activating the Nrf2/ARE pathway and exert anti-inflammatory effects by modulating cytokines such as TNF-α and IL-6. Thus, porcupine bezoar shows promise in cancer adjunctive therapy, wound healing in diabetes, and inflammation regulation [24,25,26].

However, comprehensive research into the mechanisms by which porcupine bezoar addresses immunosuppression is lacking, particularly regarding its potential to modulate the ‘gut microbiota-immune axis’ to enhance immune function. This study uses multi-omics technologies to investigate how porcupine bezoar synergistically regulates gut microbiota composition, metabolite profiles and immune markers in a cyclophosphamide-induced immunosuppressed rat model. The goal is to lay the groundwork for the application of porcupine bezoar in immune enhancement and open new avenues for research.

## 2. Results

### 2.1. Effect of Porcupine Bezoar on Body Weight, Food Intake, and Immune Organ Indices in Immunosuppressed Rats

**Figure 1:**
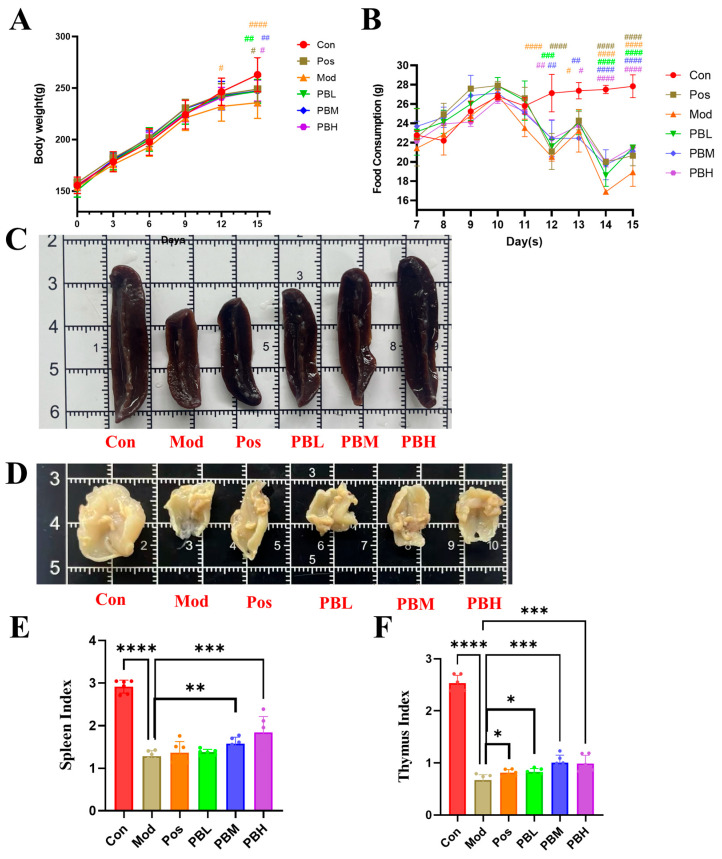
Effect of porcupine bezoar on Body Weight, Food Intake, and Spleen and Thymus Immune Indices in Immunosuppressed Rats (**A**) Body weight. (**B**) Food intake per rat. (**C**) Morphological observation of the spleen. (**D**) Morphological observation of the thymus. (**E**) Spleen index. (**F**) Thymus index. Con, blank control group; Mod, model group; Pos, positive drug group treated with Spleen Aminopeptide Oral Lyophilized Powder; PBL, low-dose porcupine bezoar group; PBM, medium-dose porcupine bezoar group; PBH, high-dose porcupine bezoar group. Body weight and food intake were analyzed by two-way repeated-measures ANOVA, whereas spleen and thymus indices were analyzed using one-way ANOVA. Statistical significance is indicated in the graphs at the relevant timepoints or endpoints. * *p* < 0.05, ** *p* < 0.01, *** *p* < 0.001, **** *p* < 0.0001 versus the Mod group. ^#^ *p* < 0.05, ^##^ *p* < 0.01, ^###^ *p* < 0.001, ^####^ *p* < 0.0001 versus the Con group.

To investigate the protective effects of PB on immune organs, we established an immunosuppressed rat model using CTX. Prior to modeling, the initial body weights of all groups were comparable, and body weight gradually increased from Day 1, with similar growth trends across all groups. However, by Day 11, when modeling commenced, the control group maintained a steady weight gain rate, while the model group showed a reduced rate of weight gain. Both the positive drug group and the PB treatment groups exhibited a similar weight growth trend, which, although slowed, showed some recovery (Figure 1A). The food intake curve showed a similar trend across all groups before modeling. After the induction of immunosuppression, the changes in food intake mirrored the weight gain pattern. The model group displayed the greatest reduction in food intake, indicating that CTX reduced food consumption and slowed growth, successfully inducing the immunosuppressed rat model (Figure 1B).

Spleen Examination: In the control group, the spleen of healthy adult rats measured approximately 2–3 cm in length, presenting a dark red color, elongated oval or crescent shape, smooth surface, and soft, fragile texture. In the model group, spleen tissue showed signs of atrophy with a decrease in weight, indicating an immunosuppressive state. The spleen surface appeared slightly rough, and the tissue became firm, suggesting damage. Both the positive drug group and PB treatment groups alleviated these pathological changes. The positive drug group exhibited a smooth surface, soft texture, and partial recovery in spleen volume. The PB treatment group showed further restoration, with the mid-dose and high-dose groups resembling the control group in terms of size, indicating a potential protective effect on the spleen (Figure 1C). Thymus Examination: In the control group, the thymus was divided into left and right lobes, with a uniform milky-white color, soft texture, and a thin, smooth capsule. In the model group, thymic tissue volume significantly reduced, with a decrease in weight, dull color, and surface adhesion, indicating an immunosuppressive condition. The positive drug group exhibited thymus volume intermediate between the control and model groups, with partial recovery, though not returning to normal levels. In the PB treatment group, the thymus also showed partial restoration, with the PBM group demonstrating significant thymic protection or repair (Figure 1D).

Compared to the control group, the spleen index (spleen weight/body weight ratio) in the model group significantly decreased, indicating that CTX induced atrophy of immune organs and impaired immune function. The positive drug group showed some recovery in spleen index, and treatment with various doses of PB resulted in a dose-dependent restoration of the spleen index. Notably, the high-dose PB group (PBH) exhibited the most significant effect (Figure 1E). Similarly, the thymus index (thymus weight/body weight ratio) followed a pattern similar to the spleen index. The Mod group showed a significant reduction in thymus index. After treatment with different doses of PB, the thymus index showed partial recovery, with the mid- and high-dose groups demonstrating comparable results (Figure 1F).

### 2.2. Effect of Porcupine Bezoar on Liver and Thymus Inflammation in CTX-Induced Immunosuppressed Rats and Its Impact on Serum Inflammatory Cytokines

**Figure 2:**
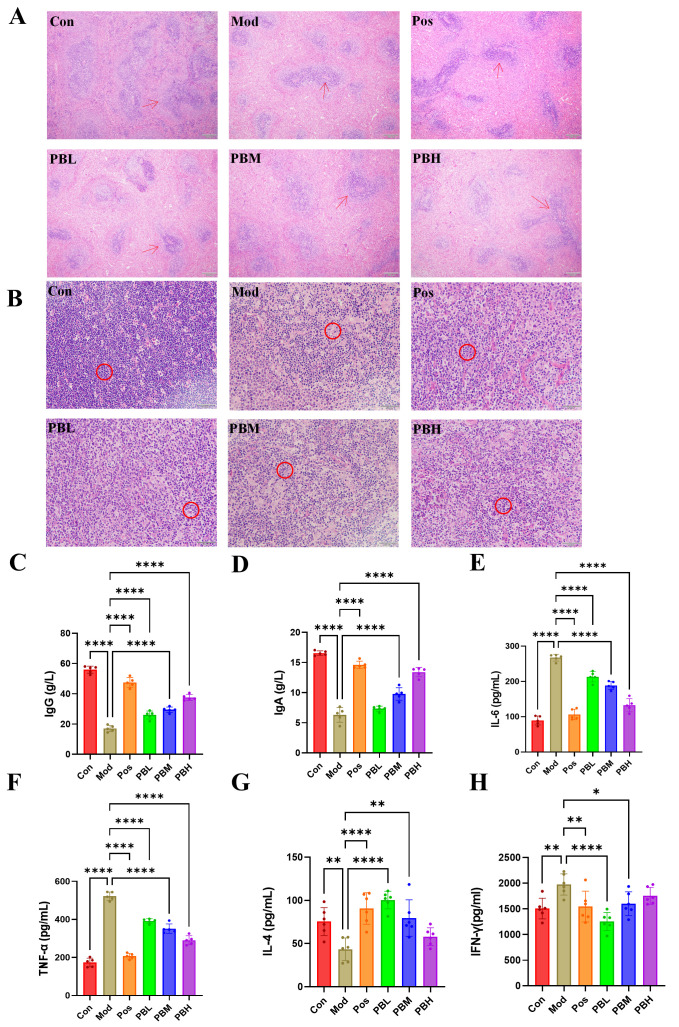
Effect of porcupine bezoar on Spleen and Thymus Tissue Structure and Inflammatory Response in CTX-Induced Rats (**A**) HE staining of rat spleen (×40), with red arrows indicating lymphoid follicles. (**B**) HE staining of rat thymus (×200), with red circles highlighting lymphocyte density. (**C**) ELISA analysis of serum IgG levels. (**D**) ELISA analysis of serum IgA levels. (**E**) ELISA analysis of serum IL-6 levels. (**F**) ELISA analysis of serum TNF-α levels. (**G**) ELISA analysis of serum IL-4 levels. (**H**) ELISA analysis of serum IFN-γ levels. Compared to the model group, * *p* < 0.05; ** *p* < 0.01; **** *p* < 0.0001.

Histopathological examination of the spleen was performed using Hematoxylin and Eosin (HE) staining. The spleen’s white pulp is composed of dense lymphoid cells that proliferate during antigen invasion, initiating a humoral immune response. The red pulp, which consists of splenic sinusoids and cords, serves as the primary site for immune cell phagocytosis. In the Con group, the spleen exhibited normal architecture with distinct boundaries between the red and white pulp, a normal ratio, large lymphoid follicles, and tightly arranged lymphocytes, showing no abnormal findings. In contrast, the Mod group displayed a significant reduction in white pulp area, an increase in the red pulp area, a decrease in the size of lymphoid follicles, and a marked reduction in lymphocyte numbers, indicating spleen damage. In the PB and Pos groups, the white pulp area was restored, with a more distinct boundary between red and white pulp and a notable increase in lymphocyte numbers, suggesting a reduction in pathological damage (Figure 2A). Thymic histological analysis revealed that the Con group exhibited normal thymus structure with closely packed lymphocytes. In the Mod group, thymic lymphocyte arrangement became looser with a reduction in density, indicating compromised thymic immune function. Conversely, the PB and Pos groups showed a significant increase in thymic lymphocyte numbers compared to the Mod group, with more densely distributed lymphocytes, partially improving the pathological damage (Figure 2B).

In the Mod group, the levels of immunoglobulins IgA and IgG were significantly reduced, indicating a decline in immune function following CTX-induced immunosuppression. In contrast, the PB treatment groups showed a dose-dependent recovery of both IgA and IgG levels compared to the Mod group, suggesting that porcupine bezoar effectively restores immune function to some extent (Figure 2C,D). Moreover, the pro-inflammatory cytokines IL-6 and TNF-α were significantly elevated in the Mod group compared to the Con group, indicating a chronic inflammatory state in the immunosuppressed rats. This heightened innate immune response, rather than providing protection, exacerbates the dysfunction of adaptive immunity. Following PB treatment, levels of both pro-inflammatory cytokines were significantly reduced in all dose groups compared to the Mod group. These results clearly demonstrate that PB can effectively modulate excessive innate immune inflammation, thereby alleviating the dysregulated immune response (Figure 2E,F).

IL-4 and IFN-γ play antagonistic roles, which underpins the core concept of Th1/Th2 balance in immunology. Compared to the Mod group, the serum levels of IL-4 were significantly elevated, while IFN-γ levels were reduced in all treatment groups at the conclusion of the study. These results indicate that PB effectively enhances the Th2-type humoral immune response (IL-4 ↑) and reverses CTX-induced immune suppression, while also inhibiting the excessive Th1-type cellular immune response (IFN-γ ↓) observed in the Mod group. This suggests that PB may promote immune homeostasis through a Th2-biased regulation of humoral immunity (Figure 2G,H).

### 2.3. Porcupine Bezoar Ameliorates CTX-Induced Intestinal Inflammation and Modulates Intestinal Permeability in Rats

**Figure 3:**
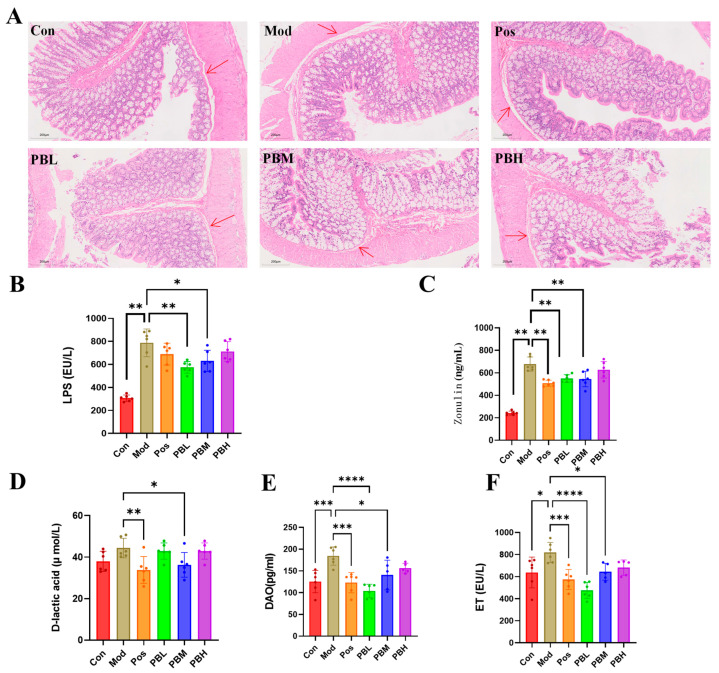
Effect of porcupine bezoar on Intestinal Inflammation and Intestinal Permeability in CTX-Induced Rats (**A**) H&E staining of distal colon sections (×200), with red arrows indicating the mucosal epithelial layer. Representative images were selected from comparable distal colon sections and are presented at the same magnification and similar tissue orientation across groups. (**B**) Serum levels of Lipopolysaccharide (LPS). (**C**) Serum levels of Zonulin. (**D**) Serum levels of D-lactic acid. (**E**) Serum levels of diamine oxidase (DAO). (**F**) Serum levels of endotoxin (ET). Compared to the model group, * *p* < 0.05; ** *p* < 0.01; *** *p* < 0.001; **** *p* < 0.0001.

Histological examination of distal colonic tissue in the Con group revealed preserved mucosal architecture, characterized by orderly epithelial cell arrangement, absence of submucosal thickening or collagen deposition, clear crypt morphology, and no obvious inflammatory cell infiltration or tissue injury. In contrast, the Mod group exhibited marked structural damage to the distal colon, including epithelial disorganization and partial detachment, evident submucosal thickening with collagen deposition, crypt distortion or loss, and inflammatory cell infiltration. Compared with the Mod group, the treatment groups showed clear histological improvement to varying degrees. These improvements were characterized by better preservation of epithelial integrity, attenuation of submucosal thickening and collagen deposition, partial restoration of crypt architecture, and reduced inflammatory cell infiltration. Among the treatment groups, PB administration showed particularly notable recovery of distal colonic histological structure (Figure 3A).

Compared with the Con group, both the Mod group and the treatment groups exhibited significantly elevated levels of Lipopolysaccharide (LPS) and Zonulin. Upon treatment completion, levels of LPS and Zonulin in the treatment groups were significantly reduced compared with the Mod group (*p* < 0.01), indicating that PB administration effectively improved intestinal permeability and enhanced intestinal barrier function (Figure 3B,C). Further analysis revealed significantly elevated levels of diamine oxidase (DAO), endotoxin (ET), and D-lactic acid in both the Mod group and the treatment groups when compared with the Con group. CTX-induced immunosuppression may disrupt intestinal barrier integrity, thereby increasing ET and D-lactic acid levels and exacerbating the risk of secondary infection. After treatment, all dosage groups showed significant reductions in DAO, ET, and D-lactic acid levels compared with the Mod group, suggesting that PB administration effectively ameliorated intestinal barrier dysfunction and strengthened intestinal barrier integrity (Figure 3D–F).

### 2.4. Non-Targeted Plasma Metabolomics: Screening of Differential Metabolites

**Figure 4:**
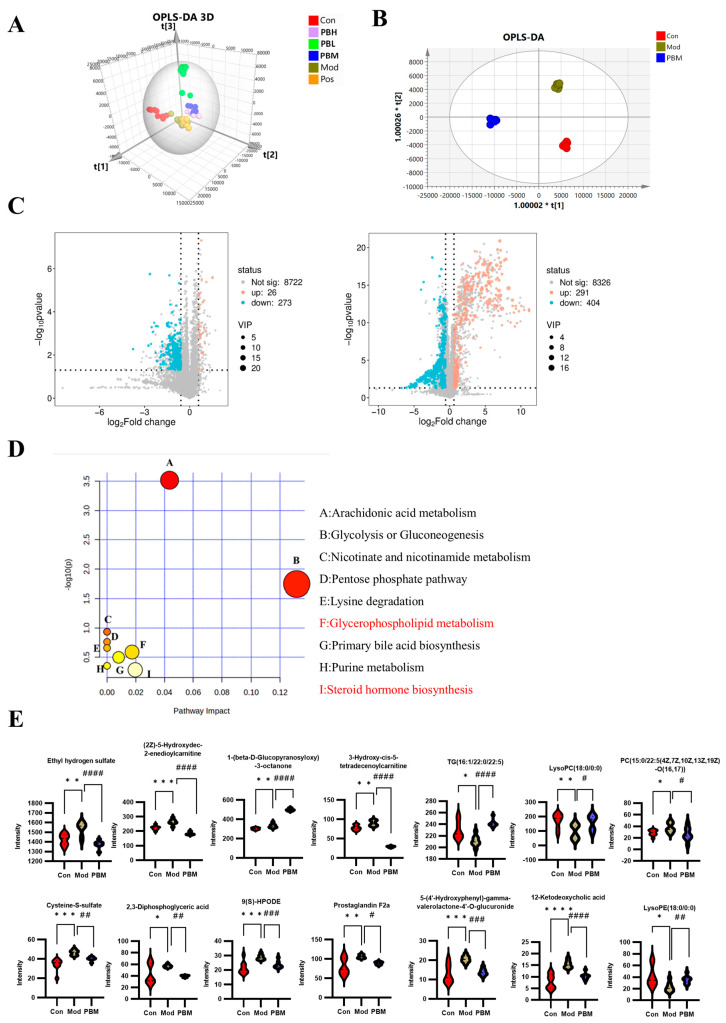
Non-targeted Plasma Metabolomics: Screening of Differential Plasma Metabolites (**A**) 3D Orthogonal partial least squares discriminant analysis (OPLS-DA) plot of plasma metabolomics for rats in each group. (**B**) OPLS-DA score plot for the positive mode: Con-Mod-PBM plasma metabolomics. (**C**) Volcano plots showing differential metabolites between Con vs. Mod and Mod vs. PBM. (**D**) Pathway enrichment analysis of differential plasma metabolites. (**E**) Statistical analysis of 14 key differential metabolites. Data are presented as mean ± SD. Comparisons between groups were made using one-way ANOVA with post hoc analysis. * *p* < 0.05, ** *p* < 0.01, *** *p* < 0.001, **** *p* < 0.0001 versus the Con group. ^#^ *p* < 0.05, ^##^ *p* < 0.01, ^###^ *p* < 0.001, ^####^ *p* < 0.0001 versus the Mod group.

To identify differential metabolites, all groups were first subjected to Orthogonal partial least squares discriminant analysis (OPLS-DA) under positive ion mode. The results revealed a distinct distribution pattern among the five groups, with noticeable variation between groups, indicating a certain degree of dispersion. Samples that clustered closely together suggested similarities in their metabolite composition and concentrations. Notably, the PBM group in the PB treatment group was found to be closer to the Con group, suggesting a potential alignment in metabolite profiles that warrants further investigation (Figure 4A).

To investigate the plasma metabolic changes associated with immune enhancement and gut health improvement by PB, statistical analysis was conducted on the Con, Mod, and PBM groups. OPLS-DA modeling was used to compare the clustering characteristics of the three groups. The results revealed clear separation between the Con, Mod, and PBM groups, with each group clustering separately. This indicates that the metabolic state of the rats in the MOD group had significantly changed, and that PB treatment induced further metabolic alterations in the rats’ plasma (Figure 4B).

Volcano plots, a type of scatter plot, combine *p*-values with fold change (FC) to visually and efficiently identify metabolites or proteins with significant differential expression. Using SIMCA-derived VIP, *p*-values, and FC values, a volcano plot analysis was performed through a bioinformatics platform. In the Con vs. Mod comparison, 26 metabolites were upregulated, while 273 metabolites were downregulated. In the Mod vs. PBM comparison, 291 metabolites were upregulated, and 404 metabolites were downregulated. A comprehensive comparison of Con, Mod, and PBM plasma metabolites led to the identification of 38 differential metabolites related to PB’s effects on immune enhancement and gut health improvement (Table 1). Among these, 14 metabolites were upregulated in the positive mode, and 24 were upregulated in the negative mode (Figure 4C).

To further investigate the upstream metabolic pathways associated with PB’s effects on immune modulation and gut health improvement, MetaboAnalyst was used for pathway analysis of the 38 differential metabolites. The results revealed that these metabolites were involved in nine key metabolic pathways, which include: (A) Arachidonic acid metabolism, (B) Glycolysis/gluconeogenesis, (C) Nicotinate/nicotinamide metabolism, (D) Pentose phosphate pathway, (E) Lysine degradation, (F) Glycerophospholipid metabolism, (G) Primary bile acid biosynthesis, (H) Purine metabolism, and (I) Steroid hormone biosynthesis (Figure 4D). Based on the *p*-values derived from the differential metabolite enrichment analysis, we hypothesize that glycerophospholipid metabolism and steroid hormone biosynthesis are key pathways in PB’s immune protection mechanism.

We performed statistical analysis on the 38 differential metabolites, identifying 14 metabolites significantly impacted by PB. Notably, PB caused the downregulation of 10 metabolites, including Ethyl hydrogen sulfate, (2Z)-5-Hydroxydec-2-enedioylcarnitine, 3-Hydroxy-cis-5-tetradecenoylcarnitine, Cysteine-S-sulfate, 2,3-Diphosphoglyceric acid, 9(S)-HPODE, Prostaglandin F2a, 5-(4′-Hydroxyphenyl)-gamma-valerolactone-4′-O-glucuronide, 12-Ketodeoxycholic acid, and PC(15:0/22:5(4Z,7Z,10Z,13Z,19Z)-O(16,17)). Additionally, four metabolites were upregulated by PB, including 1-(beta-D-Glucopyranosyloxy)-3-octanone, TG(16:1/22:0/22:5), LysoPC(18:0/0:0), and LysoPE(18:0/0:0) (Figure 4E).

### 2.5. Identification of Key Metabolic Pathways Involved in Immune Regulation Through Plasma Differential Metabolite-Associated Proteins

**Figure 5:**
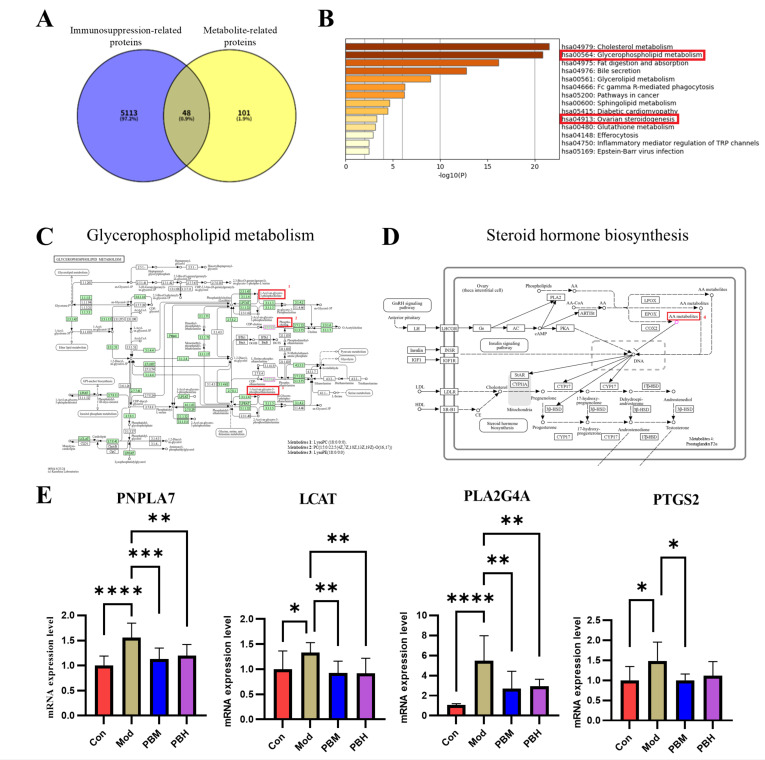
Identification of Key Metabolic Pathways Involved in Immune Modulation via Differential Plasma Metabolite-Related Proteins (**A**) Venn diagram showing the overlap between immunosuppressive-related proteins and metabolite-related proteins. (**B**) KEGG pathway enrichment analysis of intersecting targets. (**C**) Overview of the glycerophospholipid metabolism biosynthesis pathway (MetPA). (**D**) Overview of the steroid hormone biosynthesis pathway (MetPA). (**E**) Relative expression levels of key differential metabolite-related genes in the glycerophospholipid metabolism and steroid hormone biosynthesis pathways in rat spleen tissue. Data are expressed as mean ± SD. Statistical significance was determined by one-way ANOVA with post hoc tests. * *p* < 0.05, ** *p* < 0.01, *** *p* < 0.001, **** *p* < 0.0001.

To validate glycerophospholipid metabolism and steroid hormone biosynthesis as critical metabolic pathways, we conducted a protein network analysis of the 14 significant differential metabolites. Relevant proteins for these metabolites were retrieved from the HMDB and Uniprot databases. After correcting gene names in Uniprot and removing duplicates, we identified a total of 149 proteins. Further, we collected 5161 immune suppression-related proteins from the DisGeNET, OMIM, and GeneCard databases. By performing a Venn diagram analysis, we intersected the metabolite-related proteins with immune suppression-related proteins, resulting in 48 common target proteins (Figure 5A).

We performed KEGG pathway enrichment analysis of the 48 common targets using the Metascape online tool. The results revealed that both glycerophospholipid metabolism and steroid hormone biosynthesis pathways were significantly enriched among the common targets. Further pathway analysis using MetaboAnalyst and KEGG corroborated these findings, suggesting that glycerophospholipid metabolism and steroid hormone biosynthesis are likely key metabolic pathways involved in the protective effects of PB against immune suppression (Figure 5B).

According to the overview diagram generated by Metascape for glycerophospholipid metabolism and steroid hormone biosynthesis, the small molecule metabolites associated with compound 1, compound 2, compound 3, and compound 4 are as follows: LysoPC(18:0/0:0), PC(15:0/22:5(4Z,7Z,10Z,13Z,19Z)-O(16,17)), LysoPE(18:0/0:0) and Prostaglandin F2a. They may be the key differential metabolites that play a crucial role in enhancing the immune system and improving intestinal health in rats exposed to arrow pine seeds (Figure 5C,D).

Based on the genes involved in glycerophospholipid metabolism and steroid hormone biosynthesis, we further identified several pathway-related candidate genes associated with immunosuppression. In the glycerophospholipid metabolism pathway, PNPLA7 and LCAT, and in the steroid hormone biosynthesis pathway, PLA2G4A and PTGS2, were selected as key candidate genes for transcriptional validation. To further investigate whether PB intervention was associated with changes in these pathway-related genes in immunosuppressed rats, RT-qPCR was performed to assess their expression levels in spleen tissue. In the glycerophospholipid metabolism pathway, compared with the Con group, the mRNA levels of PNPLA7 and LCAT were significantly increased in the Mod group, suggesting that CTX-induced immunosuppression was accompanied by a disturbance in the transcriptional profile of genes related to glycerophospholipid metabolism. After PB treatment, the expression of both genes was partially restored toward the control level, indicating that PB may help normalize transcriptional changes associated with glycerophospholipid metabolic disturbance. In the steroid hormone biosynthesis pathway, the mRNA levels of PLA2G4A and PTGS2 were also significantly elevated in the Mod group compared with the Con group, suggesting that immunosuppression was accompanied by transcriptional alterations in genes involved in this pathway. PB treatment reduced the expression levels of these genes, indicating that PB was associated with a partial reversal of the abnormal gene expression pattern induced by CTX. Overall, these RT-qPCR findings were consistent with the metabolomic results and suggest that glycerophospholipid metabolism and steroid hormone biosynthesis may participate in the immunomodulatory effects of PB at the transcript level (Figure 5E).

### 2.6. Remodeling Effects of Porcupine Bezoar on the Gut Microbiota in Cyclophosphamide-Induced Immunosuppressed Rats

**Figure 6:**
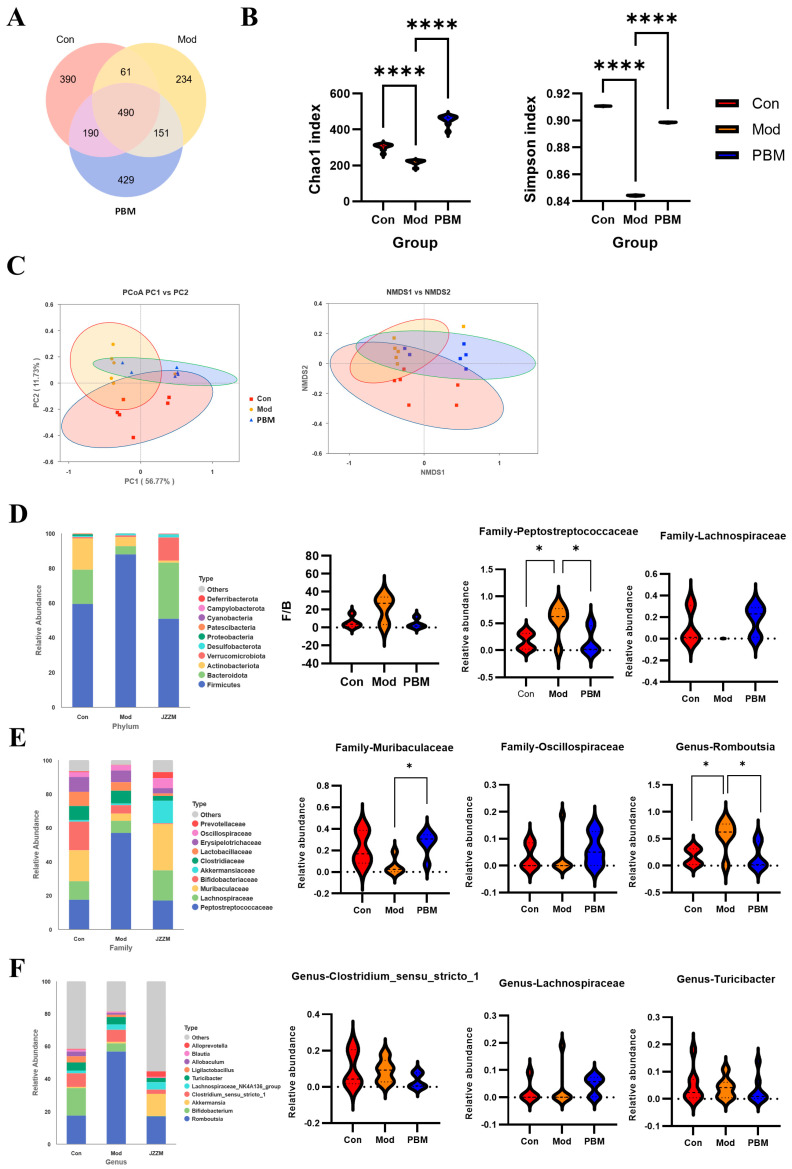
Reshaping of the Gut Microbiota in Cyclophosphamide-Induced Immunosuppressed Rats by porcupine bezoar (**A**) Venn diagram displaying the number of unique and shared features among the different samples. (**B**) Analysis of α-diversity index, representing the species diversity within each group. (**C**) Beta-diversity analysis, showing the differences in microbiota composition between groups. (**D**) Relative abundance of microbial species at the phylum level. (**E**) Relative abundance of microbial species at the class level. (**F**) Relative abundance of microbial species at the genus level. Data are expressed as mean ± SD. Statistical significance was evaluated using one-way ANOVA followed by post hoc tests. * *p* < 0.05, **** *p* < 0.0001.

To assess the similarity and overlap of amplicon sequence variant (ASV) compositions among groups, we clustered sequencing reads using a single-nucleotide–resolution approach (100% similarity threshold). Compared with traditional OTU-based methods, ASV-based profiling offers superior sensitivity and substantially reduces false-positive assignments, thereby providing more accurate and reproducible microbial community characterization. Across all samples, a total of 490 ASVs were identified. Among these, the Con and Mod groups shared 61 ASVs; the Mod and PBM groups shared 151 ASVs; and the Con and PBM groups shared 190 ASVs. This pattern indicates that cyclophosphamide markedly altered the colonic microbial consortium, whereas PB treatment partially restored microbial composition toward that of healthy controls, suggesting a significant modulatory effect of PB on gut microbial community structure (Figure 6A).

The α-diversity index reflects the richness (Chao1 index) and diversity (Simpson index) of microbial communities. Higher values indicate greater species richness, diversity, and evenness. Compared to the Con group, the PBM group showed significant increases in both Chao1 and Simpson indices, indicating an enhancement in microbial diversity and richness. In contrast, all indices were decreased in the Mod group, suggesting a reduction in microbial diversity and richness in the immunocompromised rats (Figure 6B). In the β-diversity analysis, PCoA and NMDS results were consistent, showing significant separation of gut microbiota among the groups, with clear structural differences. The microbiota structure of the PBM group was shifted away from the Mod group, approaching that of the Con group, suggesting that PB intervention partially restored the gut microbiota composition (Figure 6C).

The gut microbiota community at the phylum level is predominantly composed of *Firmicutes*, *Bacteroidetes*, and *Actinobacteria*, which together account for over 95% of the total bacterial phyla. *Bacteroidetes* are crucial for maintaining immune system stability and tolerance, promoting regulatory T cell differentiation, and inhibiting excessive inflammation. In contrast, the *Firmicutes* phylum is known to be involved in energy metabolism and immune modulation. In the Mod group, the abundance of *Firmicutes* was significantly higher compared to the Con and PBM groups, while the abundance of *Bacteroidetes* was lower in the Mod group compared to both the Con and PBM groups. Further analysis of the *Firmicutes*/*Bacteroidetes* (F/B) ratio showed no statistically significant difference among the groups. The Mod group displayed a broader distribution and an upward shift in F/B ratio, whereas the PBM group tended to show a distribution closer to that of the Con group. However, given the substantial overlap and inter-individual variability among groups, these observations should be interpreted cautiously. These results suggest that immunosuppression may lead to an imbalance in the *Firmicutes* and *Bacteroidetes* ratio, and that PB treatment may partially alleviate this imbalance (Figure 6D).

At the family level, significant differences were observed in key bacterial families such as *Peptostreptococcaceae*, *Lachnospiraceae*, *Muribaculaceae*, and *Oscillospiraceae*. In particular, the abundance of *Peptostreptococcaceae* in the Mod group was significantly higher than that in the Con and PBM groups, while the abundances of *Lachnospiraceae* and *Muribaculaceae* were lower in the Mod group compared to the other two groups. These changes suggest a potential dysregulation of the gut microbiota in immunocompromised rats, particularly affecting bacterial families related to metabolism and immune modulation. The observed shifts in these families may reflect underlying disruptions in metabolic and immune functions (Figure 6E).

At the genus level, further analysis revealed notable differences in the composition of key microbial genera. Specifically, the abundance of *Romboutsia* (*Peptostreptococcaceae*) and *Clostridium sensu stricto 1* (*Clostridiaceae*) was significantly higher in the Mod group compared to both the Con and PBM groups. Additionally, the abundance of *Turicibacter*, *Lachnospiraceae* in the Mod group was either higher or comparable to the other two groups. These changes in the relative abundance of specific genera are closely associated with dysbiosis, particularly the overgrowth of *Romboutsia*, which may negatively affect gut barrier integrity and contribute to an inflammatory response (Figure 6F).

### 2.7. Porcupine Bezoar Mediated Immune Enhancement Is Associated with the Interaction Between Gut Microbiota and Metabolite Abundance

**Figure 7:**
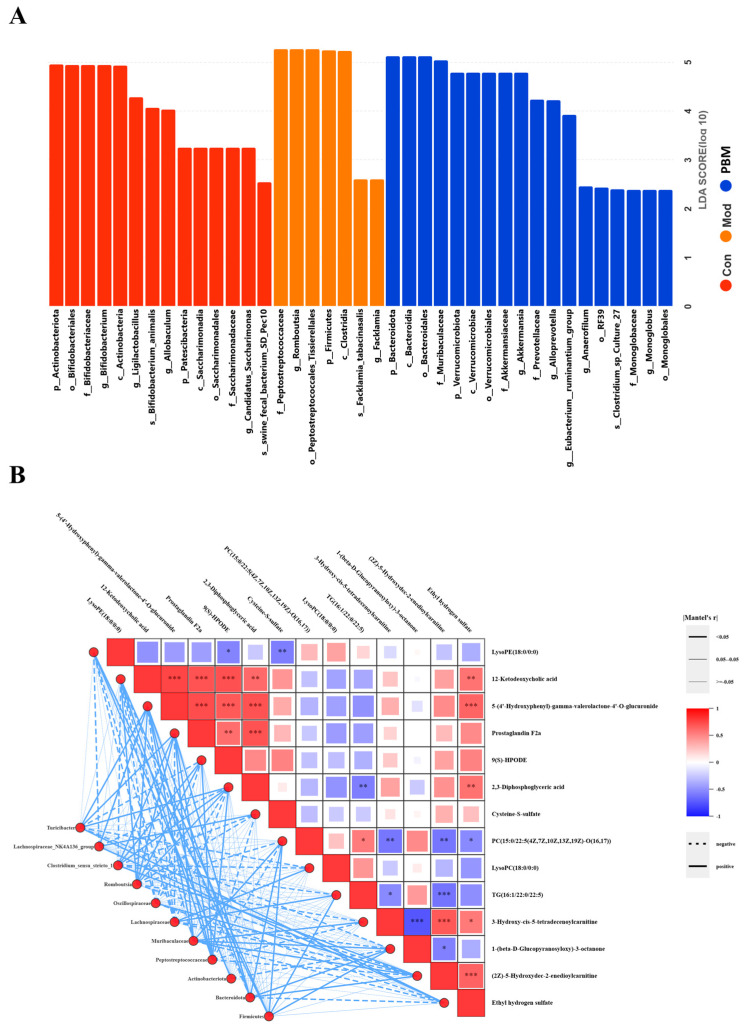
Porcupine bezoar Influences the Enrichment of Specific Gut Microbiota and Associated Differential Metabolites (**A**) LEfSe analysis of the Linear Discriminant Analysis (LDA) distribution bar chart, highlighting significant microbial taxa in different experimental groups. (**B**) Mantel test correlation analysis between important differential metabolites and gut microbiota composition. In the heatmap, the horizontal and vertical axes represent different metabolites and gut microbiota species, respectively. On the left side, microbiota species that are highly correlated with specific metabolites are linked, with the line thickness indicating the degree of significance and the line color reflecting the strength of the correlation. Statistical significance was evaluated using Mantel’s test and indicated by the line thickness and color. * *p* < 0.05, ** *p* < 0.01, *** *p* < 0.001.

To further identify microbial taxa with significant differences across groups, Linear Discriminant Analysis Effect Size (LEfSe) analysis was employed. LEfSe is a robust analytical method that combines non-parametric statistical tests with Linear Discriminant Analysis effect size, allowing for the identification of statistically significant biomarkers between groups. The LEfSe analysis LDA distribution bar plot revealed 39 distinct differentially abundant taxa, each belonging to different classifications. The results demonstrate unique microbial abundance profiles across groups at various taxonomic levels (Figure 7A).

To elucidate whether the changes in metabolite abundance associated with PB administration contribute to immune enhancement via modulation of the gut microbiota, we performed a Mantel test analysis between 14 key differential metabolites in plasma and 11 distinct gut microbiota taxa. We found that certain serum metabolites, including 12-Ketodeoxycholic acid, Prostaglandin F2α, 2,3-Diphosphoglyceric acid, 9(S)-HPODE, and Cysteine-S-sulfate, exhibited strong correlations with specific gut microbes (e.g., *Firmicutes*, *Peptostreptococcaceae*, *Romboutsia*). And metabolites such as PC(15:0/22:5(4Z,7Z,10Z,13Z,19Z)-O(16,17)), TG(16:1/22:0/22:5), and LysoPE (18:0/0:0) were strongly correlated with *Bacteroidota* and *Muribaculaceae*. Among these eight metabolites, those associated with glycerophospholipid metabolism and steroid hormone biosynthesis, such as PC(15:0/22:5(4Z,7Z,10Z,13Z,19Z)-O(16,17)), Prostaglandin F2α, and LysoPE(18:0/0:0), were linked to *Bacteroidota*, Muribaculaceae, *Firmicutes*, *Peptostreptococcaceae*, and *Romboutsia* species. Notably, *Peptostreptococcaceae* is a higher taxon of *Romboutsia*. These correlation analyses indicate that PB-induced immune enhancement and gut health improvement in rats are closely associated with the key metabolites in glycerophospholipid metabolism and steroid hormone biosynthesis pathways, particularly with *Peptostreptococcaceae* and its associated metabolites PC(15:0/22:5(4Z,7Z,10Z,13Z,19Z)-O(16,17)), Prostaglandin F2α, and LysoPE(18:0/0:0) (Figure 7B). These findings provide important insights into the mechanisms through which PB metabolites influence gut microbiota to enhance immune function in rats.

## 3. Discussion

The porcupine bezoar, with its long history of use in traditional medicine, has shown potential in antioxidant, anti-inflammatory, and anti-tumor activities. However, its capacity to enhance immune function and prevent or treat diseases remains insufficiently explored. This study aims to investigate the immunoregulatory effects of porcupine bezoar on immune-deficient rats using plasma metabolomics and 16S rDNA sequencing. We established a model of immune suppression in rats induced by cyclophosphamide, a well-established system used to simulate human immunodeficiency and assess potential therapeutic agents. CTX induces immune suppression by inhibiting DNA synthesis and cell proliferation, particularly in rapidly dividing immune cells such as lymphocytes, leading to immune dysfunction. In this study, we administered varying doses of porcupine bezoar via prophylactic gavage to examine its potential in modulating immune responses and improving immune function in CTX-induced immune-deficient rats.

Our results demonstrate that administration of porcupine bezoar significantly mitigated the effects of CTX-induced immunosuppression in rats, as evidenced by improvements in body weight and thymus and spleen immune indices. CTX treatment reduced food intake, slowed growth, and decreased the size of primary immune organs, successfully establishing a model of immune deficiency that recapitulates aspects of human immunodeficiency and serves as a validated platform for evaluating potential therapeutics. Following PB administration, rats exhibited partial restoration of body weight trajectories and immune organ indices compared with the model group. The thymus and spleen, as central immune organs, are critical for lymphocyte development and proliferation; increases in their immune indices generally reflect enhanced organ development and improved immune function. Consistent with this, we observed that serum immunoglobulin levels, key effectors of adaptive immunity produced by B lymphocytes in response to antigen stimulation, were elevated following PB treatment. Analysis of serum immunoglobulin levels revealed that IgA and IgG concentrations were lowest in the CTX-treated model group, confirming at the molecular level that cyclophosphamide-induced immunosuppression markedly impaired adaptive immune function. In contrast, all treatment groups exhibited significant increases in IgA and IgG levels, with PB administration producing a dose-dependent restoration. These findings indicate that PB partially restores humoral immunity in immunocompromised rats, supporting its potential role in enhancing systemic immune competence. Notably, IgG plays a pivotal role by binding directly to pathogens or their toxins, neutralizing their capacity to invade host cells or cause tissue damage. Collectively, these findings suggest that PB enhances both the structural integrity of immune organs and systemic adaptive immune responses in CTX-immunosuppressed rats [27,28]. Previous studies have demonstrated that impaired immune function directly activates inflammatory pathways, leading to the release of pro-inflammatory cytokines such as TNF-α and IL-6. TNF-α, primarily secreted by macrophages and monocytes, is one of the earliest and most critical mediators of the inflammatory response under physiological conditions [29]. IL-6 plays a pivotal role in immune regulation by promoting B cell differentiation and antibody production, as well as T cell activation, proliferation, and differentiation; it also modulates TNF-α expression, thereby amplifying inflammatory signaling [30,31]. Consistent with these mechanisms, serum analyses in our study revealed markedly elevated levels of TNF-α and IL-6 in the CTX-treated model group compared to controls. Administration of PB significantly normalized these pro-inflammatory cytokines relative to the model group. These findings corroborate the histopathological observations of immune organs and indicate that PB effectively mitigates CTX-induced inflammation associated with immunosuppression.

IL-4, predominantly produced by type 2 helper T cells (Th2), serves as a critical initiator of humoral and Th2-mediated immune responses and represents a major anti-inflammatory cytokine [32]. In contrast, IFN-γ, produced by activated Th1 cells and natural killer (NK) cells, is a central effector of cell-mediated immunity, and elevated levels often indicate concurrent tissue damage [33]. In the CTX-induced immunosuppressed model, IL-4 levels were lowest, whereas IFN-γ levels were elevated. Mechanistically, IFN-γ suppresses IL-4 transcription via induction of IRF-1 and IRF-2, which inhibit IL-4 promoter activity, thereby reinforcing Th1 responses [34]. Notably, PB administration restored humoral immunity by enhancing Th2-type responses while concurrently restraining excessive Th1-mediated cellular immunity. These findings suggest that PB may reestablish immune homeostasis by promoting a Th2-biased regulation of humoral immunity in immunosuppressed conditions.

Histopathological analysis of the spleen and thymus further corroborated these findings, demonstrating that PB markedly counteracts CTX-induced immunosuppression. In the Mod group, splenic white pulp area was reduced, red pulp proportion increased, lymphoid follicles were smaller, and lymphocyte counts were markedly decreased, indicating substantial splenic damage following CTX administration. In contrast, PB-treated rats exhibited increased white pulp area, clearer demarcation between red and white pulp, and higher lymphocyte density, reflecting mitigation of splenic pathology. Similarly, thymic tissue from PB-treated animals showed a significant increase in lymphocyte numbers and more compact distribution compared to the Mod group, indicating partial restoration of thymic structure and function. These observations underscore the protective and restorative effects of PB on central immune organs under immunosuppressive conditions.

To systematically elucidate the mechanisms by which PB enhances immune function, we conducted untargeted plasma metabolomics to assess alterations in small-molecule metabolites under pathological conditions and following therapeutic intervention. PCA three-dimensional (3D) plots and OPLS-DA analyses demonstrated clear separation and clustering among the Con, Mod, and PBM groups, indicating pronounced metabolic perturbations in the immunosuppressed model and modulatory effects of PB treatment. Notably, the PBM group exhibited a metabolic profile closer to the Con group than the PBH group, suggesting optimal efficacy at the medium dose; consequently, this group was selected for subsequent metabolomic analyses. Volcano plot analysis integrating *p*-values and fold change (FC) from OPLS-DA, with criteria VIP > 1 and *p* < 0.05, revealed that glycerophospholipid metabolism and steroid hormone biosynthesis pathways represent key metabolic routes through which PB exerts its immunoprotective effects. Glycerophospholipid metabolism generates key phospholipids, including phosphatidylcholine (PC) and phosphatidylethanolamine (PE), which serve as substrates for phospholipase A2-mediated arachidonic acid release, thereby contributing to inflammatory signaling. Beyond their role in inflammation, these phospholipids maintain intestinal epithelial barrier integrity and participate in MHC class II-mediated antigen presentation, essential for activating adaptive immunity. Steroid hormone biosynthesis converts cholesterol into bioactive hormones such as cortisol, aldosterone, and sex steroids. Cortisol suppresses pro-inflammatory cytokine production, including IL-6 and TNF-α, while glucocorticoids induce lymphocyte apoptosis, thereby modulating autoimmune responses. Collectively, these metabolic pathways provide a mechanistic link between PB administration, immune homeostasis, and intestinal barrier function. In total, 38 potential differential metabolites associated with immune modulation were identified. Ester compounds such as phosphatidylcholine (PC), phosphatidylglycerol (PG), phosphatidylethanolamine (PE), triglycerides (TG), and lysophosphatidylcholine (LPC) may regulate immune responses by influencing T cell proliferation, B cell activation, and macrophage function. Bile acid–derived steroids, including cholyllysine, 4-hydroxy-D4-neuroprostane, and 12-ketodeoxycholic acid, are critical for maintaining intestinal barrier integrity and modulating both local and systemic immune responses. Fatty acid–derived metabolites such as prostaglandins and 4-hydroxy-D4-neuroprostane exhibit potent anti-inflammatory and immunosuppressive activities, serving as essential endogenous mediators of immune homeostasis. Together, these metabolites provide mechanistic insight into how PB administration orchestrates immune regulation and intestinal health. Statistical analysis of the 38 identified metabolites revealed 14 key differential metabolites that were significantly modulated by PB, including 10 downregulated and 4 upregulated metabolites. Notably, PC(15:0/22:5(4Z,7Z,10Z,13Z,19Z)-O(16,17)) levels were reduced following PB administration. This oxidized phosphatidylcholine, generated from phosphatidylcholine under oxidative stress via free radical–mediated modifications, is known to drive sustained inflammatory responses and can induce apoptosis, acting as a pro-inflammatory mediator [35,36]. These findings indicate that PB mitigates pro-inflammatory signals in plasma, exerting anti-inflammatory effects. Moreover, the observed downregulation of Prostaglandin F2α, a classical pro-inflammatory mediator, further corroborates our experimental evidence that PB enhances immune function at least in part through anti-inflammatory mechanisms.

Subsequent biological network analysis of the 14 key differential metabolites revealed that glycerophospholipid metabolism and steroid hormone biosynthesis were significantly enriched among the intersecting protein targets in KEGG pathway analysis, consistent with predictions from plasma metabolomics. Integrative pathway enrichment analyses using MetaboAnalyst and KEGG suggest that these two metabolic pathways represent critical mechanisms by which PB mitigates immunosuppression. Within these pathways, the small-molecule metabolites LysoPC(18:0/0:0), PC(15:0/22:5(4Z,7Z,10Z,13Z,19Z)-O(16,17)), LysoPE(18:0/0:0), and Prostaglandin F2α appear to play pivotal roles, highlighting their potential contribution to PB-mediated immunomodulation. Prostaglandin F2α, a metabolite derived from arachidonic acid, plays a central role in regulating reproductive functions, including steroid hormone biosynthesis [37]. Based on the four key differential metabolites, we further identified genes associated with immunosuppression: PNPLA7 and LCAT within glycerophospholipid metabolism, and PLA2G4A and PTGS2 within steroid hormone biosynthesis. Notably, PNPLA7 functions as an intracellular lysophospholipase that hydrolyzes PC to generate LPC [38]. Previous studies have shown that PNPLA7 knockout in mouse liver reduces the expression of inflammation-related genes, suggesting that PNPLA7 promotes hepatic inflammatory responses [39]. Our findings extend this observation, as RT-qPCR revealed elevated PNPLA7 expression in immunosuppressed model rats, which was significantly downregulated following PB administration. These results indicate that PNPLA7 modulates lipid metabolism, and its downregulation may reduce the production of inflammation-associated lipid mediators, including prostaglandins and phospholipids, thereby contributing to the attenuation of excessive immune activation.

Lecithin–cholesterol acyltransferase (LCAT) acts as an intermediate catalytic enzyme that converts PC into LPC [40], serving as a critical link between lipid metabolism, immune regulation, and cancer progression [41]. LCAT has shown considerable potential in therapeutic interventions targeting malignancies. In our study, PC levels were reduced in the PB-treated group compared to the immunosuppressed model group, while RT-qPCR demonstrated a corresponding downregulation of LCAT expression. Notably, LPC levels increased following PB administration, suggesting that treatment not only attenuates systemic inflammation but also promotes accumulation of LPC, which itself possesses bioactive properties capable of modulating cellular functions through interactions with cell membranes. Collectively, LCAT appears to regulate the PC–LPC balance, contributing to cholesterol reverse transport and maintaining lipid metabolic homeostasis, thereby supporting immune stabilization and mitigating inflammatory responses.

PLA2G4A, a cytosolic phospholipase A2, catalyzes the hydrolysis of arachidonic acid (AA) from membrane phospholipids upon activation, representing the rate-limiting step in the production of potent pro-inflammatory mediators, including prostaglandins and leukotrienes [42,43]. Upregulation of PLA2G4A activity typically exacerbates both local and systemic inflammatory responses. In the context of CTX-induced profound immunosuppression, sustained high PLA2G4A activity likely contributes to tissue damage and inflammatory cascades. RT-qPCR analysis revealed that PB administration significantly downregulated the mRNA expression of PLA2G4A. Mechanistically, PLA2G4A releases AA to serve as a substrate for PTGS2, which converts AA into prostaglandins, thereby amplifying inflammatory signaling; this establishes a functional synergy between the two enzymes. PTGS2 (cyclooxygenase-2) catalyzes the rate-limiting conversion of AA to prostaglandin F2α (PGF2α), a pivotal mediator of inflammation capable of suppressing T-cell responses [44]. CTX-induced tissue injury and inflammatory stress elevate PTGS2 expression, promoting excessive PGF2α production and fostering a locally immunosuppressive microenvironment. In alignment with PLA2G4A findings, RT-qPCR demonstrated that the mRNA expression of PTGS2 was also significantly reduced in the PB-treated group. These findings support a transcript-level association between PB treatment and the arachidonic acid/prostaglandin-related inflammatory axis. However, because protein expression and enzyme activity were not assessed in the present study, these results should be interpreted as supportive rather than definitive mechanistic evidence.

In recent years, the gut microbiota has garnered significant attention due to its central role in host–microbe interactions and its influence on systemic immunity. Modulation of the gut microbiota has therefore emerged as a critical strategy for enhancing immune function. To investigate whether PB can ameliorate immunosuppression via the gut–immune axis, we performed histopathological analyses of distal colonic tissue. Compared with the control group, the CTX-induced model group exhibited marked disruption of colonic histological architecture, including epithelial disorganization and detachment, submucosal thickening with collagen deposition, crypt distortion, inflammatory cell infiltration, and overall tissue injury. Notably, PB administration partially restored distal colonic structure, as reflected by improved epithelial integrity, reduced submucosal thickening, clearer crypt architecture, and decreased inflammatory cell accumulation and infiltration. These findings indicate that PB mitigates CTX-induced colonic injury and support a potential role for gut microbiota modulation in its immunoprotective effects.

Assessment of blood lipopolysaccharide (LPS) and its antibodies provides an indirect measure of intestinal permeability and bacterial translocation [45]. Zonulin, a key regulator of tight junctions, can increase intestinal permeability, thereby contributing to the pathogenesis of gut-associated disorders, including inflammatory bowel disease, celiac disease, and metabolic syndrome [46,47]. In our study, PB administration significantly reduced serum levels of LPS and Zonulin compared with the model group, indicating enhanced intestinal barrier integrity. Diamine oxidase (DAO), an enzyme expressed in intestinal epithelial cells, is released into circulation upon mucosal damage, serving as a marker of barrier disruption [48]. Similarly, endotoxin (ET) can translocate into the bloodstream when the gut barrier is compromised, activating innate immune responses; elevated ET levels reflect gut-derived inflammation, and chronic exposure to LPS can trigger systemic immune activation and inflammation. D-lactate, a bacterial fermentation product, also rises in circulation when gut microbiota balance is perturbed and barrier function declines [49]. Excessive D-lactate not only reflects dysbiosis and metabolic disturbance but may further exacerbate local acidification, promoting immune dysregulation in the gut [50]. In the model group, we observed a significant elevation of DAO, ET, and D-lactate levels, indicative of impaired intestinal barrier function and disrupted immune homeostasis. Following treatment, all PB-administered groups exhibited a marked reduction in DAO, ET, and D-lactate levels compared with the model group, demonstrating that PB effectively restored intestinal barrier integrity and mitigated gut-derived immune imbalance. Collectively, these results demonstrate that PB administration improves intestinal barrier function and mitigates systemic inflammation associated with CTX-induced immunosuppression.

Subsequently, we performed 16S rDNA sequencing to characterize the gut microbiota of the rats. Comparative analysis across taxonomic levels revealed that PB’s immunomodulatory effects are closely associated with its regulation of the abundance of key microbial taxa, including *Firmicutes*, *Bacteroidetes*, *Actinobacteria*, *Peptostreptococcaceae*, *Lactobacillaceae*, *Muribaculaceae*, and *Lachnospiraceae*. In the model group, the relative abundances of *Bacteroidetes* and *Lachnospiraceae* were significantly reduced, whereas PB administration restored their levels. *Bacteroidetes* play a pivotal role in maintaining immune system stability and tolerance by promoting the differentiation of regulatory T cells and suppressing excessive inflammatory responses. *Lachnospiraceae*, a prominent family within *Firmicutes*, contributes critically to gut health and immune regulation, largely through the production of short-chain fatty acids, particularly butyrate, which enhances intestinal barrier integrity and promotes immune tolerance [51,52]. Our results demonstrated that the relative abundances of *Romboutsia* (*Peptostreptococcaceae*) and *Clostridium sensu stricto* (*Clostridiaceae*) were significantly elevated in the model group compared with the control and PB-treated groups. Overgrowth of *Romboutsia* can adversely affect intestinal barrier integrity and promote inflammatory responses, while increased *Clostridium sensu stricto* is often indicative of compromised gut barrier function and the initiation of systemic low-grade inflammation [53]. Such dysbiosis facilitates the translocation of pathogenic bacteria or their metabolites into the circulation, thereby chronically stimulating the immune system—a common pathological basis for multiple chronic diseases. Notably, Our study revealed that PB effectively suppressed the proliferation of harmful bacteria, including *Romboutsia* and *Clostridium sensu stricto*, while significantly enhancing the abundance of beneficial *Bacteroidetes*. These findings indicate that the preventive and therapeutic effects of PB against immunosuppression are closely associated with its ability to modulate detrimental gut microbes. Collectively, PB appears to restore immune competence by regulating the gut microbiota–immune axis, thereby improving host immune function under immunocompromised conditions.

Mantel test correlation analysis was performed to evaluate the associations between key plasma differential metabolites and altered gut microbiota. We found that the serum metabolite Prostaglandin F2α exhibited strong correlations with specific gut taxa, including *Firmicutes*, *Peptostreptococcaceae*, and *Romboutsia*, whereas PC(15:0/22:5(4Z,7Z,10Z,13Z,19Z)-O(16,17)), TG(16:1/22:0/22:5), and LysoPE(18:0/0:0) were strongly associated with *Bacteroidota* and *Muribaculaceae*. These metabolites are linked to gut microbes such as *Bacteroidota*, *Muribaculaceae*, *Firmicutes*, *Peptostreptococcaceae*, and *Romboutsia*, with *Peptostreptococcaceae* serving as the higher taxonomic unit of *Romboutsia*. The pronounced correlation of Prostaglandin F2α with other metabolites suggests that it plays a pivotal role within a complex immune regulatory network, particularly in orchestrating inflammation, lipid metabolism, oxidative stress, gut health, and immune homeostasis [54]. Rather than acting as a singular inflammatory mediator, Prostaglandin F2α likely interacts across multiple metabolic pathways, synergistically influencing immune and metabolic processes. Overall, our correlation analysis indicates that the immunomodulatory effects of PB and its improvement of gut health are closely associated with *Peptostreptococcaceae* and key metabolites within glycerophospholipid metabolism and steroid hormone biosynthesis pathways, including PC(15:0/22:5(4Z,7Z,10Z,13Z,19Z)-O(16,17)), Prostaglandin F2α, and LysoPE(18:0/0:0).

It is important to note that translating the immunomodulatory effects of PB observed in rat models to human applications entails several challenges. First, physiological functions, metabolic pathways, and disease progression differ between humans and rats, which may result in variations in PB efficacy between species. Therefore, future clinical studies must be carefully designed to rigorously evaluate the effectiveness of PB in humans. Second, interspecies dose translation is complex. Differences in body weight, metabolic rate, and drug clearance mean that the doses used in rat experiments cannot be directly applied to humans. Consequently, human studies will require careful dose selection, alongside comprehensive pharmacokinetic and pharmacodynamic investigations to ensure safety. Finally, given the heterogeneity among immunocompromised populations, future clinical trials should stratify participants according to the underlying causes of immunodeficiency to accurately assess the preventive and therapeutic potential of PB across different disease contexts.

## 4. Materials and Methods

### 4.1. Reagents

Porcupine bezoar, the batch used in this study (Batch No. 202406) was supplied by Miracle Medicine Sdn Bhd (Sunway South Quay, Bandar Sunway, Selangor, Malaysia) and declared to be of Indonesian origin. The material was stored at room temperature until use. Traceability documentation (Batch information, origin declaration, and research-use declaration) is provided in Supplementary File S1, and a voucher specimen has been deposited at the Institute of Traditional Chinese Medicine, Guangdong Pharmaceutical University under voucher ID PB-202406-01, Oral Lyophilized Powder of Spleen Polypeptide (Batch #: 20200539, Dalian Baili Tianhua Pharmaceutical Co., Ltd., Dalian, China), and Cyclophosphamide (Batch #: 0B364A, Baxter Oncology GmbH, Halle/Westfalen, Germany). The water used in the experiments was sourced from Hangzhou Wahaha Group Co., Ltd. (Hangzhou, China). The following reagents were obtained from Servicebio Technology Co., Ltd. (Wuhan, China): Paraformaldehyde Fixative Solution, Hematoxylin Staining Solution, Hematoxylin Differentiation Solution, Hematoxylin Bluing Solution, and Eosin Staining Solution. Enzyme-Linked Immunosorbent Assay (ELISA) kits were purchased from Shanghai Enzyme-linked Biotechnology Co., Ltd. (Shanghai, China). FastPure Cell/Tissue Total RNA Isolation Kit V2 version RC112, HiScript III RT SuperMix for qPCR (gDNA wiper) kit version R323 and ChamQ Universal SYBR qPCR Master Mix Kit were purchased from Vazyme Biotech Co., Ltd. (Nanjing, China).

### 4.2. Porcupine Bezoar Material and Quality Assessment

An independent third-party quality control (QC) assessment of the porcupine bezoar (porcupine dates) powder was conducted by SGS (Malaysia) Sdn Bhd, and the test reports are provided in Supplementary File S2. The tested material was labeled as “Porcupine Dates Powder (Extraction Version)” (sample ID: AH19377; job No.: H&N/2025-03-24-020). The QC panel included an amino acid profile, microbiological limits (TAMC, TYMC, bile-tolerant Gram-negative bacteria; British Pharmacopoeia Appendix XVIB), absence of key pathogens (*Escherichia coli*, *Staphylococcus aureus*, *Salmonella*), and heavy metal levels (As, Cd, Pb, Hg; AOAC 2013.06). An additional SGS microbiological report (sample ID: AH72788; job No.: H&N/2026-01-26-008) confirmed microbiological limits and pathogen absence. Collectively, these data support traceability and basic quality/safety compliance of the crude drug material used in this study, although they do not substitute for batch-specific chromatographic fingerprinting of the Indonesian material. Key QC results are summarized in Supplementary File Table S1; full reports are provided in Supplementary File S2.

### 4.3. Chemical Composition Framework of Porcupine Bezoar

Previous studies have profiled porcupine bezoar extracts using LC–MS/MS and GC–MS, providing a reference framework for this complex mixture. LC–ESI–MS/MS of tannin-enriched fractions reported gallic acid and galloyl derivatives (polygalloyl glucoses), while GC–MS of aqueous extracts repeatedly detected fatty acids/esters and bile-acid/steroid-related components (e.g., tetradecanoic acid, ursodeoxycholic acid, and a cholest-5-en-3-ol derivative). Phytochemical screening further suggested tannins as a major phenolic class. These reports are cited as supportive background only and do not constitute confirmed identification of constituents in the specific batch used in the present study. Future studies should incorporate batch-specific HPLC/MS fingerprinting and targeted constituent analysis to strengthen reproducibility and chemical standardization. Reported chemical classes/representative constituents of porcupine bezoar extracts from previous studies in Supplementary File Table S2.

### 4.4. Animals and Experimental Design

Sixty SPF male Sprague-Dawley rats, aged 30–35 days and weighing 100–130 g, were obtained from the Guangdong Provincial Medical Laboratory Animal Center. The animals were housed under SPF conditions at the Guangdong Pharmaceutical University Animal Experiment Center. The facility operates under the licenses SYXK (Yue) 2022-0125 and SCXK (Yue) 2022-0002. Animals were allowed *ad libitum* access to food and water and were maintained in a controlled environment with a 12 h light/dark cycle, a temperature of 22 ± 2 °C, and a relative humidity of 50 ± 10%, in accordance with experimental standards. The study was registered under the protocol number SPF2022565, and all procedures were approved by the Guangdong Pharmaceutical University Animal Ethics Committee (gdpulacspf2022565), following the institution’s ethical guidelines for animal experimentation.

After a 3-day acclimatization period, the rats were randomly divided into six groups (*n* = 10 per group) based on similar body weight: Blank control group (Con), model group (Mod), positive drug group (Pos) treated with Spleen Aminopeptide Oral Lyophilized Powder, a spleen-derived immunomodulatory preparation composed mainly of peptides and nucleotide-related substances extracted from fresh pig spleen, low-dose porcupine bezoar group (PBL), medium-dose porcupine bezoar group (PBM), and high-dose porcupine bezoar group (PBH). The PB dosing regimen was designed with reference to a putative human intake of 0.4 g/day for a 60-kg adult. The medium dose (PBM) was selected to approximate the corresponding rat-equivalent dose, while the low-dose (PBL) and high-dose (PBH) groups were set as approximately half-dose and two-fold dose-ranging groups, respectively. The PBL, PBM, and PBH groups received oral gavage of porcupine bezoar at doses of 2.1 mg/100 g/day, 4.2 mg/100 g/day, and 8.4 mg/100 g/day, respectively. The Pos group received an oral gavage of Spleen Aminopeptide Oral Lyophilized Powder (0.042 mg/100 g/day), while the Con and Mod groups received an equal volume of water. All gavage treatments were administered at a dose of 1 mL/100 g/day for 15 days. On days 11, 12, and 13, except for the Con group, all other groups received a 3-day regimen of 3.5 mg/100 g/day cyclophosphamide (CTX) via intraperitoneal injection to induce an immunosuppressed rat model. This treatment protocol lasted for 15 days, during which the rats’ behavior, body condition, and health status were closely monitored for any abnormalities. The body weight of each rat was recorded every three days to ensure accurate dosing, and food intake was monitored daily. Rats had free access to food and water throughout the study period, and general health indicators were observed.

**Figure 8:**
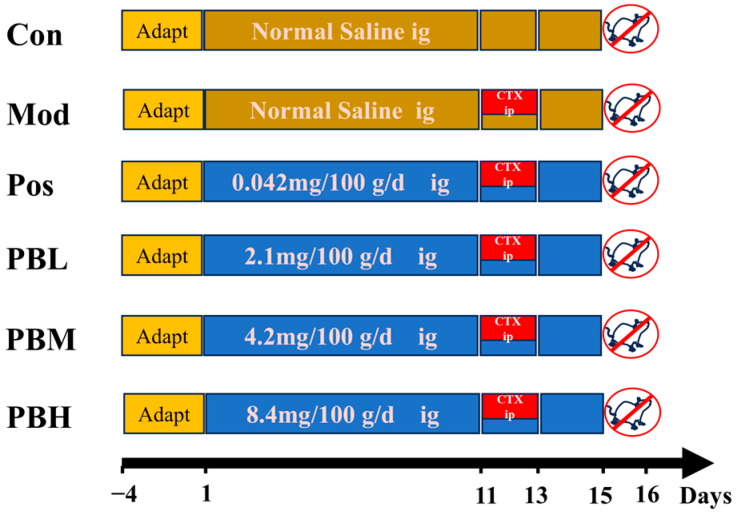
Schematic representation of experimental design. Con, blank control group; Mod, model group; Pos, positive drug group (Spleen Aminopeptide Oral Lyophilized Powder); PBL, low-dose porcupine bezoar group; PBM, medium-dose porcupine bezoar group; PBH, high-dose porcupine bezoar group; CTX, cyclophosphamide (*n* = 10).

On day 15, 24 h after the last dose of treatment, the rats were fasted for 12 h but allowed free access to water. Their body weights were measured and recorded. All rats were anesthetized with isoflurane, and blood was collected from the abdominal aorta. The blood was left to stand at 4 °C for 1 h and then centrifuged at 3500 rpm for 15 min. The supernatant was collected and stored at −80 °C for subsequent analysis. Following blood collection, the spleen, thymus, heart, intestine, and intestinal contents were quickly excised. Half of the spleen, thymus, heart, and colon tissues were fixed in 4% formalin for histopathological examination, while the remaining tissues were immediately frozen at −80 °C for further analysis. The intestinal contents were also frozen at −80 °C for additional evaluation (Figure 8).

### 4.5. Detection of Biological-Related Indicators in Serum

After blood collection from the abdominal aorta, samples were stored in EDTA anticoagulant tubes and allowed to stand at 4 °C for 1 h. The samples were then centrifuged at 3500 rpm for 15 min, and the supernatant was collected. The serum levels of immunoglobulins (IgA, IgG), diamine oxidase (DAO), endotoxin (ET), D-lactic acid, and cytokines (TNF-α, IL-4, IL-6, and γ-IFN) were measured. All assays were performed following the manufacturer’s instructions provided with the ELISA kits from Shanghai Enzyme-linked Biotechnology Co., Ltd.

### 4.6. Histological Analysis of Thymus and Spleen and Calculation of Organ Indices

Fresh thymus and spleen tissues were carefully excised and rinsed multiple times with PBS buffer. The tissues were then fixed in 4% paraformaldehyde solution at room temperature for 24 h. Following fixation, the samples were embedded in paraffin, sectioned, and stained with hematoxylin and eosin (H&E) for histological examination. Tissue morphology was observed and photographed to assess pathological changes. The remaining thymus and spleen tissues were snap-frozen in liquid nitrogen and stored at −80 °C for further analysis.

At the end of the experiment on day 15, rats were weighed to determine their final body weight. After blood collection from the abdominal aorta, the spleen and thymus were carefully excised, and excess blood was blotted with filter paper. The organs were then weighed using an electronic balance (sensitivity: 0.1 mg). The organ index was calculated as the ratio of the organ weight to the final body weight (mg/g), as follows:

Thymus index = Thymus weight (mg)/Body weight (g)

Spleen index = Spleen weight (mg)/Body weight (g)

### 4.7. Intestinal Tissue Collection and Histological Observation

For histological observation, a fixed segment of the distal colon was collected from each rat as the region of interest. After gentle removal of luminal contents with PBS, the tissue was fixed in 4% paraformaldehyde at room temperature for 24 h, embedded in paraffin, sectioned, and stained with hematoxylin and eosin (H&E). Histological evaluation focused on epithelial integrity, submucosal changes, crypt architecture, inflammatory cell infiltration, and overall tissue injury. Representative images were captured at the same magnification, and the images shown for each group were selected from comparable distal colon sections with a similar orientation relative to the mucosal surface. The remaining jejunum and colon tissues were snap-frozen in liquid nitrogen and stored at −80 °C for further analysis.

### 4.8. Plasma Metabolomics

Plasma samples were retrieved from a −80 °C freezer and thawed at 4 °C. The samples were then centrifuged at 3000 rpm for 5 min at 4 °C. A 200 µL aliquot of the supernatant was transferred to a 1.5 mL centrifuge tube, and 600 µL of methanol was added. The mixture was vortexed for 1 min and incubated at 4 °C for 1 h. After incubation, the supernatant was centrifuged at 14,000 rpm for 15 min at 4 °C. The resulting supernatant was filtered through a 0.22 μm organic membrane for subsequent analysis.

Chromatographic separation was performed using an Acquity UPLC BEH C18 column (2.1 mm × 100 mm, 1.7 μm, Waters, Milford, MA, USA). The mobile phase consisted of 0.1% formic acid in water (A) and methanol (B), with a flow rate set to 0.3 mL/min. The column temperature was maintained at 35 °C, and the injection volume was 2 μL. Raw liquid chromatography-mass spectrometry (LC-MS) data were processed using PeakView 1.2 for deconvolution, followed by peak selection, calibration, and area normalization. Metabolite features were annotated based on accurate mass matching, tandem mass spectrometry data, and the Human Metabolome Database (HMDB).

Univariate statistical analysis was performed using one-way analysis of variance (ANOVA) to determine the *p*-values for all metabolites. Multivariate analysis was conducted using SIMCA software (version 14.0, Umetrics, Umea, Sweden), including principal component analysis (PCA) and orthogonal partial least squares discriminant analysis (OPLS-DA). Differential metabolites were identified based on a variable importance in projection (VIP) score > 1 and a *p*-value < 0.05. Pathway analysis of selected differential metabolites was performed using MetaboAnalyst 5.0, with pathways significantly associated with differential metabolites being selected based on a *p*-value < 0.05 or pathway impact values > 0.2.

### 4.9. Reverse Transcription Quantitative Polymerase Chain Reaction (RT-qPCR)

Total RNA was isolated from frozen spleen tissues using the FastPure Cell/Tissue Total RNA Isolation Kit V2 (RC112), following the manufacturer’s instructions. Complementary DNA (cDNA) was synthesized using the HiScript III RT SuperMix for qPCR (gDNA wiper) kit (R323). Quantitative real-time PCR was performed using the ChamQ Universal SYBR qPCR Master Mix Kit on a 7500 Fast Real-Time PCR System (Applied Biosystems, Thermo Fisher Scientific, Waltham, MA, USA). The primer sequences used for amplification are listed in Table 2. The thermal cycling conditions were as follows: initial denaturation at 95 °C for 10 min; 40 cycles of denaturation at 95 °C for 15 s, annealing at 60 °C for 30 s, and extension at 72 °C for 30 s. GAPDH was used as the internal reference gene. Relative gene expression levels were calculated using the 2^−ΔΔCt^ method.

### 4.10. Gut Microbiota Analysis

The gut was carefully excised, and the colonic contents were collected and placed in sterilized EP tubes. The samples were snap-frozen in liquid nitrogen and stored at −80 °C for subsequent analysis. The composition of the gut microbiota was analyzed through 16S rRNA gene sequencing.

### 4.11. Statistical Analysis

Statistical analysis and graphing were performed using GraphPad Prism software (version 8.0, GraphPad Software, San Diego, CA, USA). Experimental data are expressed as mean ± standard deviation (SD). Before statistical comparisons, data normality was assessed using the Shapiro–Wilk test. For normally distributed single-timepoint data, comparisons between two groups were performed using an independent-samples *t*-test, whereas comparisons among multiple groups were analyzed using one-way analysis of variance (ANOVA) followed by appropriate post hoc tests. For body weight and food consumption, which were measured repeatedly over time, two-way repeated-measures ANOVA was used to evaluate the effects of treatment group, time, and their interaction, followed by appropriate multiple-comparison testing where applicable. Data that did not conform to a normal distribution were analyzed using the corresponding non-parametric tests. Mantel correlation analysis was performed based on Pearson’s correlation coefficient. A value of *p* < 0.05 was considered statistically significant.

## 5. Conclusions

**Figure 9:**
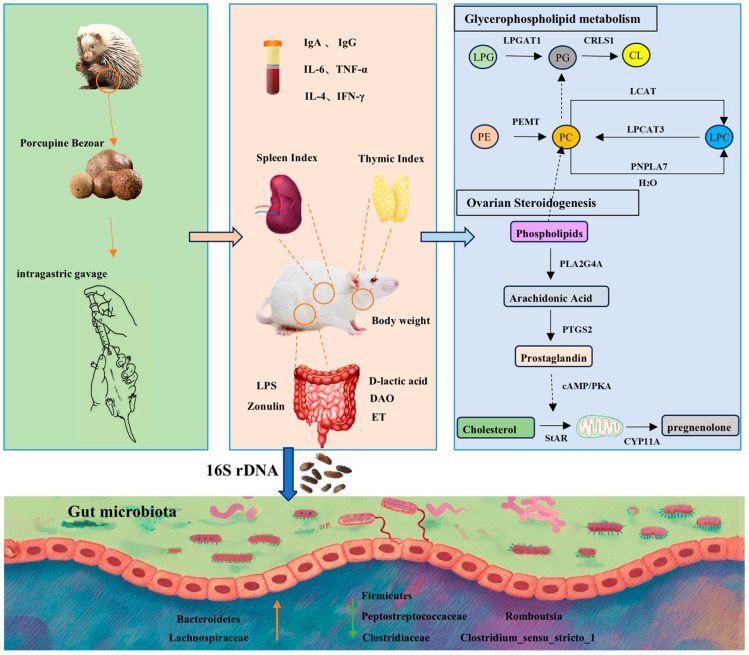
Mechanistic Summary of the Immunomodulatory Effects of porcupine bezoar (PB) in Rats. LPG: Lysophosphatidylglycerol; PG: Phosphatidylglycerol; CL: Cardiolipin; PC: Phosphatidylcholine; LPC: Lysophosphatidylcholine; PE: Phosphoethanolamine.

In summary, this study demonstrates that PB effectively reverses cyclophosphamide-induced immunosuppression in rats, ameliorating reductions in body weight, immune organ indices, and inflammatory markers, while improving histopathological alterations in the spleen and thymus. Further analyses integrating intestinal permeability indicators, plasma metabolomics, and gut microbiome profiling revealed that PB attenuates intestinal inflammatory cell infiltration, enhances gut barrier integrity, and improves drug absorption and utilization. Integrated metabolomic and RT-qPCR analyses suggest that PB may modulate host immunity through glycerophospholipid metabolism and steroid hormone biosynthesis, as reflected by altered levels of pathway-related metabolites and corresponding transcript-level changes. Evidenced by the reduction of pro-inflammatory metabolites, including Prostaglandin F2α, phosphatidylcholine PC(15:0/22:5(4Z,7Z,10Z,13Z,19Z)-O(16,17)), and 9(S)-HPODE, alongside normalization of associated gene expression (PNPLA7, LCAT, PLA2G4A, and PTGS2). Further protein-level and functional validation will be required to confirm these mechanisms. Additionally, PB reshapes the gut microbial community, increasing the abundance of beneficial taxa such as *Bacteroidota* and *Lachnospiraceae*, while reducing harmful taxa including Romboutsia and *Clostridium_sensu_stricto_1*, thereby partially shifting the gut microbiota toward a profile closer to that of the control group. Collectively, these findings elucidate the mechanistic basis by which PB enhances immune function, offering novel insights for developing preventive and therapeutic strategies for immunocompromised populations. Moreover, this work provides a theoretical foundation and practical guidance for the clinical application of PB in immune-related interventions (Figure 9).

## Figures and Tables

**Table 1:** Detailed information on the differential metabolites.

No.	Description	HMDB ID	*m*/*z*	RT (min)	Adducts	Formula	Intensity in CON	Intensity in MOD	Intensity in PBM
1	Picolinic acid	HMDB0002243	124.0864	34.10	M+H	C_6_H_5_NO_2_	610,450.13 ± 35,777.56	661,453.38 ± 33,384.25	391,951.25 ± 22,024.72
2	Ethyl hydrogen sulfate	HMDB0031233	149.0235	33.71	M+Na	C_2_H_6_O_4_S	1425,859.50 ± 57,243.16	1540,721.88 ± 69,480.70	1375,963.38 ± 39,088.12
3	Linolenelaidic acid	HMDB0030964	279.2311	32.19	M+H	C_18_H_30_O_2_	251,561.88 ± 75,256.11	174,515.13 ± 67,710.65	117,769.25 ± 27,803.80
4	(2Z)-5-Hydroxydec-2-enedioylcarnitine	HMDB0241092	301.1406	33.57	M+H	C_17_H_29_NO_7_	223,454.50 ± 14,995.18	263,646.25 ± 19,105.21	184,340.50 ± 9518.78
5	1-(beta-D-Glucopyranosyloxy)-3-octanone	HMDB0031315	324.2169	25.12	M+NH_4_	C_22_H_26_O	302,826.75 ± 11,291.77	331,691.13 ± 20,004.25	498,790.00 ± 12,135.98
6	Tetradeca-7,9,11-trienedioylcarnitine	HMDB0241420	337.1671	33.73	M+H	C_21_H_33_NO_6_	189,599.50 ± 69,727.43	123,603.75 ± 38,957.60	78,395.88 ± 11,521.60
7	Cervonoyl ethanolamide	HMDB0013627	395.2192	34.90	M+Na	C_24_H_36_O_3_	267,809.88 ± 93,793.04	147,653.63 ± 41,875.98	57,610.38 ± 8215.64
8	(9S,10E,12Z)-9-Hydroperoxyoctadeca-10,12-dienoylcarnitine	HMDB0241821	397.2142	34.9	M+H	C_25_H_45_NO_6_	110,893.13 ± 41,614.29	60,832.25 ± 17,817.30	23,551.75 ± 4385.74
9	3-Hydroxy-cis-5-tetradecenoylcarnitine	HMDB0013330	408.3093	34.29	M+Na	C_21_H_39_NO_5_	77,993.38 ± 6511.33	89,820.50 ± 7001.12	29,244.25 ± 1628.53
10	TG(16:1/22:0/22:5)	HMDB0048537	482.4055	32.13	M+2H	C_63_H_110_O_6_	229,971.88 ± 15,597.17	211,250.00 ± 9371.79	241,487.63 ± 6371.90
11	LysoPC(18:1(11Z)/0:0)	HMDB0010385	522.3559	25.61	M+H	C_26_H_52_NO_7_P	107,828.50 ± 46,973.65	41,927.50 ± 23,388.80	199,663.25 ± 66,837.76
12	LysoPC(18:0/0:0)	HMDB0010384	524.3607	25.95	M+H	C_26_H_54_NO_7_P	173,631.25 ± 49,968.61	97,115.25 ± 49,921.44	161,907.63 ± 50,862.48
13	LysoPI(18:2(9Z,12Z)/0:0)	HMDB0240597	579.2937	25.12	M+H−H_2_O	C_27_H_49_O_12_P	374,090.63 ± 33,245.18	410,580.00 ± 25,476.13	307,976.38 ± 14,258.68
14	PC(15:0/22:5(4Z,7Z,10Z,13Z,19Z)-O(16,17))	HMDB0285878	808.5822	34.90	M+H	C_45_H_78_NO_9_P	28,611.88 ± 5119.93	37,437.13 ± 8192.00	26,568.75 ± 11,635.72
15	L-Pipecolic acid	HMDB0000716	128.0346	1.83	M−H	C_6_H_11_NO_2_	38,018.75 ± 8228.34	26,228.13 ± 5866.12	43,621.75 ± 11,478.87
16	Cysteine-S-sulfate	HMDB0000731	235.926	24.28	M+Cl	C_3_H_7_NO_5_S_2_	34,308.00 ± 6315.69	46,623.00 ± 2570.49	40,202.75 ± 1962.36
17	N-Acetylhistidine	HMDB0032055	242.0805	0.97	M+FA−H	C_8_H_11_N_3_O_3_	95,793.88 ± 27,764.05	69,010.13 ± 12,622.69	109,981.63 ± 24,866.48
18	3,4-Dihydroxyphenylglycol O-sulfate	HMDB0001474	248.9742	37.24	M−H	C_8_H_10_O_7_S	84,143.63 ± 15,686.41	99,245.63 ± 3324.79	89,808.75 ± 3243.14
19	2,3-Diphosphoglyceric acid	HMDB0001294	264.9681	37.2	M−H	C_3_H_8_O_10_P_2_	42,791.00 ± 17,037.94	57,012.00 ± 2332.22	39,407.75 ± 1471.65
20	Threonylphenylalanine	HMDB0029068	265.1489	16.08	M−H	C_13_H_18_N_2_O_4_	16,399.75 ± 8836.44	46,801.75 ± 7135.29	27,432.75 ± 5216.35
21	12(13)Ep-9-KODE	HMDB0013623	309.1736	23.73	M−H	C_18_H_30_O_4_	125,689.00 ± 30,195.69	166,586.63 ± 9198.57	134,816.50 ± 7908.95
22	9(S)-HPODE	HMDB0006940	311.2229	17.10	M−H	C_18_H_32_O_4_	21,490.25 ± 4324.25	28,752.50 ± 1732.82	23,560.63 ± 2411.63
23	9,12,13-TriHOME	HMDB0004708	329.2332	12.35	M−H	C_18_H_34_O_5_	31,276.88 ± 12,313.17	20,349.25 ± 1428.56	33,462.13 ± 1101.96
24	Prostaglandin F2a	HMDB0001139	353.1995	25.89	M−H	C_20_H_34_O_5_	79,960.38 ± 21,217.52	106,397.75 ± 4391.91	89,034.50 ± 3638.08
25	4-Hydroxy-D4-neuroprostane	HMDB0012777	357.2271	18.83	M−H	C_22_H_32_O_5_	46,070.38 ± 6849.15	61,514.63 ± 4231.52	52,022.63 ± 4966.86
26	5-(4′-Hydroxyphenyl)-gamma-valerolactone-4′-O-glucuronide	HMDB0059992	363.2174	20.04	M−H	C_20_H_28_O_6_	12,875.63 ± 4760.05	20,589.63 ± 1709.36	13,886.88 ± 1771.96
27	12-Ketodeoxycholic acid	HMDB0000328	371.2425	25.3	M−H_20_−H	C_24_H_38_O_4_	7936.88 ± 3079.54	15,424.00 ± 1656.40	10,234.00 ± 1383.17
28	20-Carboxy-leukotriene B4	HMDB0006059	385.2237	18.82	M+F	C_20_H_30_O_6_	57,238.50 ± 9335.88	81,372.25 ± 5213.67	62,909.50 ± 6777.67
29	N-Palmitoyl Alanine	HMDB0241919	397.2295	27.33	M−H	C_19_H_37_NO_3_	55,339.63 ± 9802.79	71,298.25 ± 2731.37	64,306.25 ± 4035.89
30	Beta-Alanyl-CoA	HMDB0006805	418.0399	14.21	M−2H	C_24_H_41_N_8_O_17_P_3_S	19,851.25 ± 3676.37	34,200.50 ± 12,656.38	21,487.38 ± 3828.48
31	LysoPE(18:0/0:0)	HMDB0011130	462.2966	27.56	M−H_20_−H	C_23_H_48_NO_7_P	41,334.63 ± 18,046.93	22,657.75 ± 8123.95	35,567.75 ± 6703.76
32	Glycocholic acid	HMDB0000138	464.3006	11.41	M−H	C_26_H_43_NO_6_	166,250.88 ± 61,792.82	79,722.50 ± 30,279.14	148,588.00 ± 50,632.19
33	LysoPC(14:1(9Z)/0:0)	HMDB0010380	532.2879	11.41	M−H+HCOONA	C_22_H_44_NO_7_P	12,794.38 ± 7802.44	5751.25 ± 2378.24	13,765.00 ± 4913.36
34	Cholyllysine	HMDB0242379	557.3344	32.91	M−H	C_30_H_52_N_2_O_6_	8314.88 ± 6011.84	1717.50 ± 538.07	7606.88 ± 728.04
35	1-Oleoyl-sn-glycero-3-phospho-D-myo-inositol(1-)	HMDB0242160	597.3078	26.25	M−H	C27H50O12P	30,510.50 ± 7090.95	20,420.13 ± 7480.31	33,485.63 ± 9300.89
36	Urobilin	HMDB0004160	635.3362	25.63	M+FA−H	C33H42N4O6	50,207.63 ± 17,506.71	35,722.75 ± 7540.01	47,838.25 ± 11,472.01
37	Glycocholate glucuronide	HMDB0341324	680.2601	24.06	M+K−2H	C32H53NO12	26,419.00 ± 10,313.18	35,280.13 ± 3108.61	29,122.63 ± 4506.72
38	PC(MonoMe(9,5)/DiMe(9,5))	HMDB0061466	850.5617	35.23	M−H	C47H83NO10P	934,802.13 ± 243,124.33	674,369.88 ± 154,724.30	1178,200.13 ± 144,270.21

**Table 2:** Primer sequences for quantitative real-time PCR.

Gene	Forward Primer (5′–3′)	Reverse Primer (5′–3′)
PNPLA7	AGAGAAGATGTTGCAGGACCAG	AGTCAGCATAGGTTTCCTTGGG
LPCAT3	GACAGGAACTCCTTGTCCTCTG	CCCTTCACCAGCTTCATGTAGT
LCAT	TACCAAAACCAGGATACCCAGC	TCCAGCCTGGCTTTCCATTATT
CRLS1	TAGCTGGGCTAACGGATTTGTT	ATCAGCAAGTGGATCAAGAGCA
LPGAT1	GCTCAGATGATGTGGCTGATGGATC	GCTGTTGGTCACGATAGGCTCTTC
PLA2G4A	CTAATGGCCTTGGTGAGTGACT	GAGCCCACTGTCTACAACATGA
StAR	GGGAGCTCCTACAGACATATGC	GTGTTGCTTCCAGTTGAGAACC
PTGS2	ACTGTACCCGGACTGGATTCTA	CACATTGTAAGTTGGTGGGCTG
CYP11A1	CCCTGGTGACAATGGTTGGATA	CTTTCCTCCAGGCATCTGAACT

## Data Availability

The original contributions presented in this study are included in the article and Supplementary Materials. Further inquiries can be directed to the corresponding authors.

## Supplementary Materials

The following supporting information can be downloaded at https://www.mdpi.com/article/10.3390/ph19040563/s1, File S1: Figure S1: Reported chemical classes/representative constituents of porcupine bezoar extracts from previous studies; Table S1: Summary of third-party quality control results for porcupine bezoar powder. Table S2: Reported chemical classes/representative constituents of porcupine bezoar extracts from previous studies; File S2: Research sample submission document (traceability information); File S3: SGS test report (third-party QC).

## Abbreviations

The following abbreviations are used in this manuscript:

ASV	Amplicon sequence variant
CL	Cardiolipin
Con	Blank control group
CTX	Cyclophosphamide
DAO	Diamine Oxidase
ELISA	Enzyme-Linked Immunosorbent Assay
ET	Endotoxin
F/B ratio	Firmicutes to Bacteroidetes ratio
FC	Fold change
HE	Hematoxylin and eosin
HMDB	Human Metabolome Database
IgA	Immunoglobulin A
IgG	Immunoglobulin G
IL-4	Interleukin-4
IL-6	Interleukin-6
KEGG	Kyoto Encyclopedia of Genes and Genomes
LCAT	Lecithin-Cholesterol Acyltransferase
LDA	Linear Discriminant Analysis
LEfSe	Linear Discriminant Analysis Effect Size
LPC	Lysophosphatidylcholine
LPG	Lysophosphatidylglycerol
LPS	Lipopolysaccharide
MetPA	Metabolism biosynthesis pathway
Mod	Model group
OPLS-DA	Orthogonal partial least squares discriminant analysis
PB	Porcupine bezoar
PBH	High-dose porcupine bezoar group
PBL	Low-dose porcupine bezoar group
PBM	Medium-dose porcupine bezoar group
PC	Phosphatidylcholine
PCA	Principal component analysis
PE	Phosphoethanolamine
PG	Phosphatidylglycerol
PLA2G4A	Phospholipase A2, Group IVA
PNPLA7	Patatin-like Phospholipase Domain Containing 7
Pos	Positive drug group
PTGS2	Prostaglandin-endoperoxide Synthase 2
RT-qPCR	Reverse transcription quantitative polymerase chain reaction
SD	Standard deviation
TG	Triglyceride
Th1	T-helper cell type 1
Th2	T-helper cell type 2
VIP	Variable importance
