# Supplementary material for: The Immunomodulatory Effects of Porcupine Bezoar on Cyclophosphamide-Induced Immunosuppression in Rats
Source: Pharmaceuticals (Basel). 2026 Apr 1;19(4):563. doi: 10.3390/ph19040563 (PMC13119076; doi:10.3390/ph19040563)
Supplement: Supplementary file 1 [file pharmaceuticals-19-00563-s001.zip › Supplementary file S1 Figure S1 and Tables S1-S2.pdf]

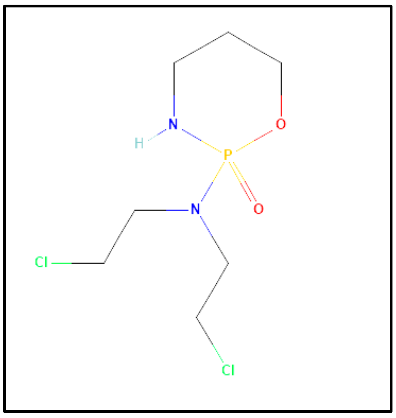

Figure S1 Reported chemical classes/representative constituents of porcupine bezoar extracts from previous studies. Chemical structure of cyclophosphamide (CTX). Molecular formula: $C_7H_{15}Cl_2N_2O_2P$

Table S1 Summary of third-party quality control results for porcupine bezoar powder.

Category	Parameter	Result	Unit	Method / Standard	Report (Sample ID; Job No.)
Sample info	Sample marking	Porcupine Dates Powder (Extraction Version)	–	–	AH19377; H&N/2025-03-24-020
Heavy metals	Arsenic (As)	0.077	mg/kg	AOAC 2013.06	AH19377; H&N/2025-03-24-020
	Cadmium (Cd)	0.013	mg/kg	AOAC 2013.06	AH19377; H&N/2025-03-24-020
	Lead (Pb)	0.26	mg/kg	AOAC 2013.06	AH19377; H&N/2025-03-24-020
	Mercury (Hg)	N.D. (<0.01)	mg/kg	AOAC 2013.06	AH19377; H&N/2025-03-24-020
Microbiological limits	Total aerobic microbial count (TAMC)	5.6 × 10^4	cfu/g	British Pharmacopoeia, Appendix XVIB	AH19377; H&N/2025-03-24-020
	Total combined yeast & mould count (TYMC)	6.0 × 10^2	cfu/g	British Pharmacopoeia, Appendix XVIB	AH19377; H&N/2025-03-24-020
	Bile-tolerant Gram-negative bacteria	N.D. (<10)	MPN/g	British Pharmacopoeia, Appendix XVIB	AH19377; H&N/2025-03-24-020
Pathogens	*Escherichia coli* (in 1 g)	Absent	–	British Pharmacopoeia, Appendix XVIB	AH19377; H&N/2025-03-24-020
	Staphylococcus aureus (in 1 g)	Absent	–	British Pharmacopoeia, Appendix XVIB	AH19377; H&N/2025-03-24-020
	Salmonella (in 25 g)	Absent	–	AOAC 2016.01 (MDA 2)	AH19377; H&N/2025-03-24-020
Amino acids (selected)	Alanine	983	mg/100 g	LFOD-TST-SOP-8512	AH19377; H&N/2025-03-24-020
	Arginine	1007	mg/100 g	LFOD-TST-SOP-8512	AH19377; H&N/2025-03-24-020
	Aspartic acid (incl. asparagine)	1568	mg/100 g	LFOD-TST-SOP-8512	AH19377; H&N/2025-03-24-020
	Glutamic acid (incl. glutamine)	2662	mg/100 g	LFOD-TST-SOP-8512	AH19377; H&N/2025-03-24-020
	Leucine	1368	mg/100 g	LFOD-TST-SOP-8512	AH19377; H&N/2025-03-24-020
	Valine	1040	mg/100 g	LFOD-TST-SOP-8512	AH19377; H&N/2025-03-24-020

Category	Parameter	Result	Unit	Method / Standard	Report (Sample ID; Job No.)
Additional SGS report (micro)	TAMC	1.2×10^5	cfu/g	British Pharmacopoeia, Appendix XVIB	AH72788; H&N/2026-01-26-008
	TYMC	4.2×10^3	cfu/g	British Pharmacopoeia, Appendix XVIB	AH72788; H&N/2026-01-26-008
	Bile-tolerant Gram-negative bacteria	N.D. (<10)	MPN/g	British Pharmacopoeia, Appendix XVIB	AH72788; H&N/2026-01-26-008
	Salmonella spp. (in 25 g)	Absent	–	British Pharmacopoeia, Appendix XVIB	AH72788; H&N/2026-01-26-008
	E. coli / *S. aureus* (in 1 g)	Absent / Absent	–	British Pharmacopoeia, Appendix XVIB	AH72788; H&N/2026-01-26-008

N.D., not detected (below the stated detection limit). Full SGS reports are provided in Supplementary File S2.

Table S2 Reported chemical classes/representative constituents of porcupine bezoar extracts from previous studies.

Study (Author, year)	Sample/Extract	Analytical method	Reported chemical classes / representative constituents
Khan et al., Antioxidants (2019)	PB aqueous extract	GC–MS	Major compounds detected included ursodeoxycholic acid, cholest-5-en-3-ol (3β)-, carbonochloridate, pentadecyl acrylate, stearic acid, and other lipid-related constituents (e.g., 1-dodecanol, amides/esters).
Khan et al., Biomedicine & Pharmacotherapy (2019)	PB-A / PB-B aqueous extracts	GC–MS	PB-A major components included dilauryl thiodipropionate, pentadecyl acrylate, 5,10-diethoxy-2,3,7,8-tetrahydro-1H, lauryl 3-mercaptopropionate; PB-B major components included ursodeoxycholic acid, cholest-5-en-3-ol (3β)-, carbonochloridate, stearic acid, and related compounds.
Khan et al., Journal of Ethnopharmacology (2020)	PB-A / PB-B / PB-C aqueous extracts (different sources/locations)	GC–MS	Across three PB aqueous extracts, 10 similar tentative compounds were reported; major putative constituents included dilauryl thiodipropionate, tetradecanoic acid, D-(+)-glucuronic acid γ-lactone, and distearyl thiodipropionate.
Yew et al., Pharmacognosy Magazine (2019)	Tannin-enriched fractions from black date (BD) and powdery date (PD) extracts	LC–ESI–MS/MS	Gallic acid and galloyl derivatives (polygalloyl glucoses) were identified, including penta-O-galloyl-β-D-glucose (PGG), tetragalloyl glucose, trigalloyl glucose, digalloyl glucose; gallic acid was also reported.
Lim/Yew et al., Pharmacognosy Research (2017)	Methanol extracts of grassy date (GD), black date (BD), powdery date (PD)	Phytochemical screening (qualitative tests)	Reported presence of hydrolysable tannins, cardiac glycosides, and terpenoids in all three; flavonoids present in BD and PD.

This table summarizes previously reported constituents/classes as background reference only.
