# Supplementary material for: The Immunomodulatory Effects of Porcupine Bezoar on Cyclophosphamide-Induced Immunosuppression in Rats
Source: Pharmaceuticals (Basel). 2026 Apr 1;19(4):563. doi: 10.3390/ph19040563 (PMC13119076; doi:10.3390/ph19040563)
Supplement: Supplementary file 1 [file pharmaceuticals-19-00563-s001.zip › Supplementary file S2 Research sample submission document (traceability information).pdf]

Research sample submission document (traceability information)

Miracle Medicine Sdn Bhd (1074325-W)
F-01-10, Sunway Geo Avenue, Jalan Lagoon Selatan,
Sunway South Quay, Bandar Sunway, 47500 Subang Jaya, Selangor.
Tel: +6011-2233 2828

## RESEARCH SAMPLE SUBMISSION DOCUMENT

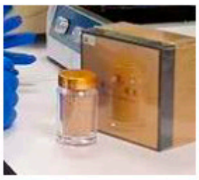

### 1. Sample Batch Information Sheet

**Product Name:** Porcupine Bezoar
**Sample Form:** Powder

**Batch Number:** 202406

**Net Weight:** 20g

**Storage Condition:** room temperature

**Intended Use:**
Research Use Only (Not for human consumption)

**Sample Provided Date:** 2024/6/14

### 2. Sample Origin Declaration

This sample is supplied by:

**Company:** Miracle Medicine Sdn Bhd
**Country of Origin:** Indonesia

Miracle Medicine Sdn Bhd (1074325-W)
F-01-10, Sunway Geo Avenue, Jalan Lagoon Selatan,
Sunway South Quay, Bandar Sunway, 47500 Subang Jaya, Selangor.
Tel: +6011-2233 2828

3. Research Use Declaration

This sample is provided solely for scientific research purposes.

It is NOT intended for:

- Human consumption
- Clinical use
- Therapeutic application

Authorized Signature

**Authorized Person:** Lim Kien Seng
**Position:** Company Director

Signature:

Date: 2024/6/14
