# Supplementary material for: The Immunomodulatory Effects of Porcupine Bezoar on Cyclophosphamide-Induced Immunosuppression in Rats
Source: Pharmaceuticals (Basel). 2026 Apr 1;19(4):563. doi: 10.3390/ph19040563 (PMC13119076; doi:10.3390/ph19040563)
Supplement: Supplementary file 1 [file pharmaceuticals-19-00563-s001.zip › Supplementary file S3 SGS test report (third-party QC).pdf]

SGS test report (third-party QC)

SGS

SAMM 077

TEST REPORT: HNSA/250550929-AH19377 **REPORTED DATE:** 30-May-2025

CUSTOMER: **MIRACLE MEDICINE SDN BHD**
F-01-10,
SUNWAY GEO AVENUE,
JALAN LAGOON SELATAN,
SUNWAY SOUTH QUAY, BANDAR SUNWAY, 47500 SUBANG JAYA, SELANGOR.

The following sample(s) was/were submitted and identified by applicant as:

**SAMPLE MARKING**	:	PORCUPINE DATES POWDER (EXTRACTION VERSION)
**SAMPLING DATE**	:	-
**SAMPLE DESCRIPTION**	:	ONE (1) SAMPLE OF POWDER
**CUSTOMER REF.**	:	-
**SAMPLE ID**	:	AH19377
**SGS JOB NO.**	:	H&N / 2025-03-24-020
**SAMPLE CONDITION**	:	AMBIENT TEMPERATURE
**SAMPLE RECEIVED**	:	24-March-2025
**TESTING PERIOD**	:	24-March-2025 to 28-May-2025
**TEST RESULTS**	:	-PLEASE REFER TO NEXT PAGE(S)-
**REMARKS**	:	1) The test result of heavy metals indicated with symbol (*) were externally provided by SGS Vietnam based on Test Report No. 0000894480.<br>2) The test parameters indicated with symbol (*) were externally provided by SGS Vietnam based on Test Report No. 0000887590.
**NOTES**		-N.D. = Not Detected

SIGNED FOR AND ON BEHALF OF
SGS (MALAYSIA) SDN BHD

FELICIA NG YEE CHIAN
TECHNICAL SPECIALIST
IKM NO. M/3434/6304/12

SIGNED FOR AND ON BEHALF OF
SGS (MALAYSIA) SDN BHD

TAN EE SAN
OPERATIONS SECTION HEAD - MICRO
FOOD ANALYST NO. MJMM0304

(Stamps: SGS (MALAYSIA) SDN. BHD. SGS 10871-T)

Test Report Form No.: SGS/TR/HN/001, Ver: 1.0, Effective Date: 10/02/2021

This document is issued by the Company subject to its General Conditions of Service printed overleaf, available on request or accessible at https://www.sgs.com/en/terms-and-conditions and, for electronic format documents, subject to Terms and Conditions for Electronic Documents at https://www.sgs.com/en/terms-and-conditions/terms-e-document. Attention is drawn to the limitation of liability, indemnification and jurisdiction issues defined therein. Any holder of this document is advised that information contained hereon reflects the Company's findings at the time of its intervention only and within the limits of Client's instructions, if any. The Company's sole responsibility is to its Client and this document does not exonerate parties to a transaction from exercising all their rights and obligations under the transaction documents. This document cannot be reproduced except in full, without prior written approval of the Company. Any unauthorized alteration, forgery or falsification of the content or appearance of this document is unlawful and offenders may be prosecuted to the fullest extent of the law. Unless otherwise stated the results shown in this test report refer only to the sample(s) tested and such sample(s) are retained for 7 days only for perishable food samples, 30 days only for non-perishable food samples, and 90 days only for fats, oils and feed samples from date of report.

SGS (Malaysia) Sdn.Bhd.
(Company No. 10871-T)
Lot 4, Persiaran Jubli Perak,Seksyen 22, 40300 Shah Alam, Selangor Darul Ehsan, Malaysia.
t +6(03) 7627 0080 f +6(03) 7627 0082 www.sgs.com

Member of the SGS Group (SGS SA)

TEST REPORT: HNSA/250550929-AH19377 **REPORTED DATE:** 30-May-2025

CUSTOMER : MIRACLE MEDICINE SDN BHD

SAMPLE ID : AH19377 - PORCUPINE DATES POWDER (EXTRACTION VERSION)

TEST RESULTS :

TEST PARAMETER	UNIT	TEST METHOD	RESULT
4-Hydroxy proline*	mg/100g	LFOD-TST-SOP-8512	N.D.(<2.6)
Alanine*	mg/100g	LFOD-TST-SOP-8512	983
Arginine*	mg/100g	LFOD-TST-SOP-8512	1007
Aspartic acid (including Asparagine)*	mg/100g	LFOD-TST-SOP-8512	1568
Cystine (sum of Cystine and Cysteine, ex	mg/100g	LFOD-TST-SOP-8512	385
Glutamic acid (including Glutamine)*	mg/100g	LFOD-TST-SOP-8512	2662
Glycine*	mg/100g	LFOD-TST-SOP-8512	687
Histidine*	mg/100g	LFOD-TST-SOP-8512	348
Isoleucine*	mg/100g	LFOD-TST-SOP-8512	741
Leucine*	mg/100g	LFOD-TST-SOP-8512	1368
Lysine*	mg/100g	LFOD-TST-SOP-8512	700
Methionine*	mg/100g	LFOD-TST-SOP-8512	475
Phenylalanine*	mg/100g	LFOD-TST-SOP-8512	895
Proline*	mg/100g	LFOD-TST-SOP-8512	648
Serine*	mg/100g	LFOD-TST-SOP-8512	1059
Threonine*	mg/100g	LFOD-TST-SOP-8512	696
Tyrosine*	mg/100g	LFOD-TST-SOP-8512	467
Valine*	mg/100g	LFOD-TST-SOP-8512	1040
Total Aerobic Microbial Count	cfu/g	British Pharmacopoeia, Microbiological Examination of Non-sterile Products, Volume V, Appendix XVIB	5.6×10^{4}
Total Combined Yeast and Mould Count	cfu/g	British Pharmacopoeia, Microbiological Examination of Non-sterile Products, Volume V, Appendix XVIB	6.0×10^{2}
Bile-Tolerant Gram-Negative Bacteria	MPN/g	British Pharmacopoeia, Microbiological Examination of Non-sterile Products, Volume V, Appendix XVIB	N.D.(<10)

SIGNED FOR AND ON BEHALF OF
SGS (MALAYSIA) SDN BHD

(SGS (MALAYSIA) SDN. BHD. 10871-T)

FELICIA NG YEE CHIAN
TECHNICAL SPECIALIST
IKM NO. M/3434/6304/12

SIGNED FOR AND ON BEHALF OF
SGS (MALAYSIA) SDN BHD

(SGS (MALAYSIA) SDN. BHD. 10871-T)

TAN EE SAN
OPERATIONS SECTION HEAD - MICRO
FOOD ANALYST NO. MJMM0304

Test Report Form No.: SGS/TR/HN/001, Ver: 1.0, Effective Date: 10/02/2021

This document is issued by the Company subject to its General Conditions of Service printed overleaf, available on request or accessible at https://www.sgs.com/en/terms-and-conditions and, for electronic format documents, subject to Terms and Conditions for Electronic Documents at https://www.sgs.com/en/terms-and-conditions/terms-e-document. Attention is drawn to the limitation of liability, indemnification and jurisdiction issues defined therein. Any holder of this document is advised that information contained hereon reflects the Company's findings at the time of its intervention only and within the limits of Client's instructions, if any. The Company's sole responsibility is to its Client and this document does not exonerate parties to a transaction from exercising all their rights and obligations under the transaction documents. This document cannot be reproduced except in full, without prior written approval of the Company. Any unauthorized alteration, forgery or falsification of the content or appearance of this document is unlawful and offenders may be prosecuted to the fullest extent of the law. Unless otherwise stated the results shown in this test report refer only to the sample(s) tested and such sample(s) are retained for 7 days only for perishable food samples, 30 days only for non-perishable food samples, and 90 days only for fats, oils and feed samples from date of report.

SGS (Malaysia) Sdn.Bhd.
(Company No. 10871-T)
Lot 4, Persiaran Jubli Perak,Seksyen 22, 40300 Shah Alam, Selangor Darul Ehsan, Malaysia.
t +6(03) 7627 0080 f +6(03) 7627 0082 www.sgs.com

TEST REPORT: HNSA/250550929-AH19377 **REPORTED DATE:** 30-May-2025

CUSTOMER : MIRACLE MEDICINE SDN BHD

SAMPLE ID : AH19377 - PORCUPINE DATES POWDER (EXTRACTION VERSION)

TEST RESULTS :

TEST PARAMETER	UNIT	TEST METHOD	RESULT
Escherichia coli in 1g	-	British Pharmacopoeia, Microbiological Examination of Non-sterile Products, Volume V, Appendix XVIB	Absent
Staphylococcus aureus in 1g	-	British Pharmacopoeia, Microbiological Examination of Non-sterile Products, Volume V, Appendix XVIB	Absent
Arsenic*	mg/kg	AOAC 2013.06	0.077
Cadmium*	mg/kg	AOAC 2013.06	0.013
Lead*	mg/kg	AOAC 2013.06	0.26
Mercury*	mg/kg	AOAC 2013.06	N.D.(<0.01)
Tryptophan*	mg/100g	LFOD-TST-SOP-8524.	22
Salmonella in 25g	-	AOAC 2016.01 (Molecular Detection Assay 2)	Absent

SIGNED FOR AND ON BEHALF OF
SGS (MALAYSIA) SDN BHD

FELICIA NG YEE CHIAN
TECHNICAL SPECIALIST
IKM NO. M/3434/6304/12

SIGNED FOR AND ON BEHALF OF
SGS (MALAYSIA) SDN BHD

TAN EE SAN
OPERATIONS SECTION HEAD - MICRO
FOOD ANALYST NO. MJMM0304

*** End of test report ***

Test Report Form No.: SGS/TR/HN/001, Ver: 1.0, Effective Date: 10/02/2021

This document is issued by the Company subject to its General Conditions of Service printed overleaf, available on request or accessible at https://www.sgs.com/en/terms-and-conditions and, for electronic format documents, subject to Terms and Conditions for Electronic Documents at https://www.sgs.com/en/terms-and-conditions/terms-e-document. Attention is drawn to the limitation of liability, indemnification and jurisdiction issues defined therein. Any holder of this document is advised that information contained hereon reflects the Company's findings at the time of its intervention only and within the limits of Client's instructions, if any. The Company's sole responsibility is to its Client and this document does not exonerate parties to a transaction from exercising all their rights and obligations under the transaction documents. This document cannot be reproduced except in full, without prior written approval of the Company. Any unauthorized alteration, forgery or falsification of the content or appearance of this document is unlawful and offenders may be prosecuted to the fullest extent of the law. Unless otherwise stated the results shown in this test report refer only to the sample(s) tested and such sample(s) are retained for 7 days only for perishable food samples, 30 days only for non-perishable food samples, and 90 days only for fats, oils and feed samples from date of report.

SGS (Malaysia) Sdn.Bhd. (Company No. 10871-T) | Lot 4, Persiaran Jubli Perak,Seksyen 22, 40300 Shah Alam, Selangor Darul Ehsan, Malaysia.
t +6(03) 7627 0080 f +6(03) 7627 0082 www.sgs.com

Member of the SGS Group (SGS SA)
